# Cellular automata imbedded memristor-based recirculated logic in-memory computing

**DOI:** 10.1038/s41467-023-38299-7

**Published:** 2023-05-10

**Authors:** Yanming Liu, He Tian, Fan Wu, Anhan Liu, Yihao Li, Hao Sun, Mario Lanza, Tian-Ling Ren

**Affiliations:** 1 School of Integrated Circuits, Tsinghua University, 100084 Beijing, China; 2 Beijing National Research Center for Information Science and Technology (BNRist), Tsinghua University, 100084 Beijing, China; 3 Weiyang College, Tsinghua University, 100084 Beijing, China; 4 Physical Science and Engineering Division, King Abdullah University of Science and Technology (KAUST), Thuwal, Saudi Arabia

**Keywords:** Electronic devices, Electrical and electronic engineering

## Abstract

Memristor-based circuits offer low hardware costs and in-memory computing, but full-memristive circuit integration for different algorithm remains limited. Cellular automata (CA) has been noticed for its well-known parallel, bio-inspired, computational characteristics. Running CA on conventional chips suffers from low parallelism and high hardware costs. Establishing dedicated hardware for CA remains elusive. We propose a recirculated logic operation scheme (RLOS) using memristive hardware and 2D transistors for CA evolution, significantly reducing hardware complexity. RLOS’s versatility supports multiple CA algorithms on a single circuit, including elementary CA rules and more complex majority classification and edge detection algorithms. Results demonstrate up to a 79-fold reduction in hardware costs compared to FPGA-based approaches. RLOS-based reservoir computing is proposed for edge computing development, boasting the lowest hardware cost (6 components/per cell) among existing implementations. This work advances efficient, low-cost CA hardware and encourages edge computing hardware exploration.

## Abstract (web summary)

Designing a full-memristive circuit for different algorithm remains a challenge. Here, the authors propose a recirculated logic operation scheme using memristive hardware and 2D transistors for cellular automata, supporting multiple algorithms with a 79-fold cost reduction compared to FPGA.

## Introduction

Cellular automata (CA) is a distinguished model that can be used to study system behavior and complex phenomena. CA not only conducts as mathematical computation models, but is also an effective medium to simulate the natural phenomena^1^ and systems^2^. When studying complex systems, CA is an efficient computing platform for its self-replication and self-organizing characteristics. As a ubiquitous and massively parallel computational model proposed by Von Neumann, CA has been used in many fields, including natural evolution^3^, cryptography^4^, image processing^5^, theoretical biology^6^, physics and microstructure modeling^7^.

CA is typically implemented in software, which demands high hardware costs. Consequently, numerous studies focus on hardware realization of CA. Popular implementation methods include very large-scale integration (VLSI) CMOS circuits^8^ and field-programmable gate array (FPGA)^9, 10^. VLSI results in a specific circuit configuration, limiting flexibility in converting different CA transition rules. While FPGA allows circuit reconfiguration, it incurs higher hardware costs. A brief schematic of CA FPGA implementation is in Supplementary Fig. 1. Thus, CA hardware realization requires a new design ensuring low cost and high flexibility for implementing CA transition rules.

In recent years, memristive circuits have emerged as low-cost, high-performance solutions for implementing in-memory computing. Various algorithm applications using memristive devices, such as reservoir computing^11–13^, neural signal analysis^14^, and convolutional neural networks^15,16^, have been realized. However, basic operations in memristive devices primarily involve matrix multiplication or logic operations. Matrix multiplication in memristive circuits is often used to accelerate data-intensive tasks like artificial neural networks^17^. Memristor-based logic operations typically design logic circuits, such as adders^18,19^ or logic gates^20,21^. Some CA transition rules can be transformed into corresponding Boolean functions, suggesting CA implementation using memristive circuits. Itoh et al. presented CA in networks of memristors mathematically, applying them to various scenarios^22^. The scheme uses charge stored in the memristor for calculations, but size reduction is limited due to capacitance scaling limitations^23^. Georgios Ch. Sirakoulis’s group proposed circuit-level design and modeling of a memristor-based CA computing array, with applications including shortest-path problems, pseudo-random number generation, bio-medical applications, and the game of life^24–28^. Their design used traditional logic gates for rule-switching logic, with hardware costs similar to FPGA implementation. Previous approaches for CA implementation with memristive circuits have not fully utilized memristor’s in-memory computing functions. There is potential for computing systems that combine CA’s structural simplicity and parallelism with memristors’ unique in-memory computing properties.

In this work, we implement a recirculated logic operation scheme (RLOS) on memristive circuitry to realize CA. This scheme combines the memory and computing characteristics of memristors, resulting in extremely low hardware costs. As a demonstration, an equivalent Turing machine based on rule 110 elementary cellular automata (ECA) has been selected to illustrate each operation during each step of evolution. Furthermore, the entire ECA, majority classification algorithm, and edge detection algorithm have been verified under RLOS. The results show that RLOS has lower hardware costs (about 2–79 times reduction) compared to FPGA implementation. Additionally, reservoir computing based on RLOS has been proposed, exhibiting lower data movement. This work opens up new opportunities for memristor applications.

**Fig. 1:**
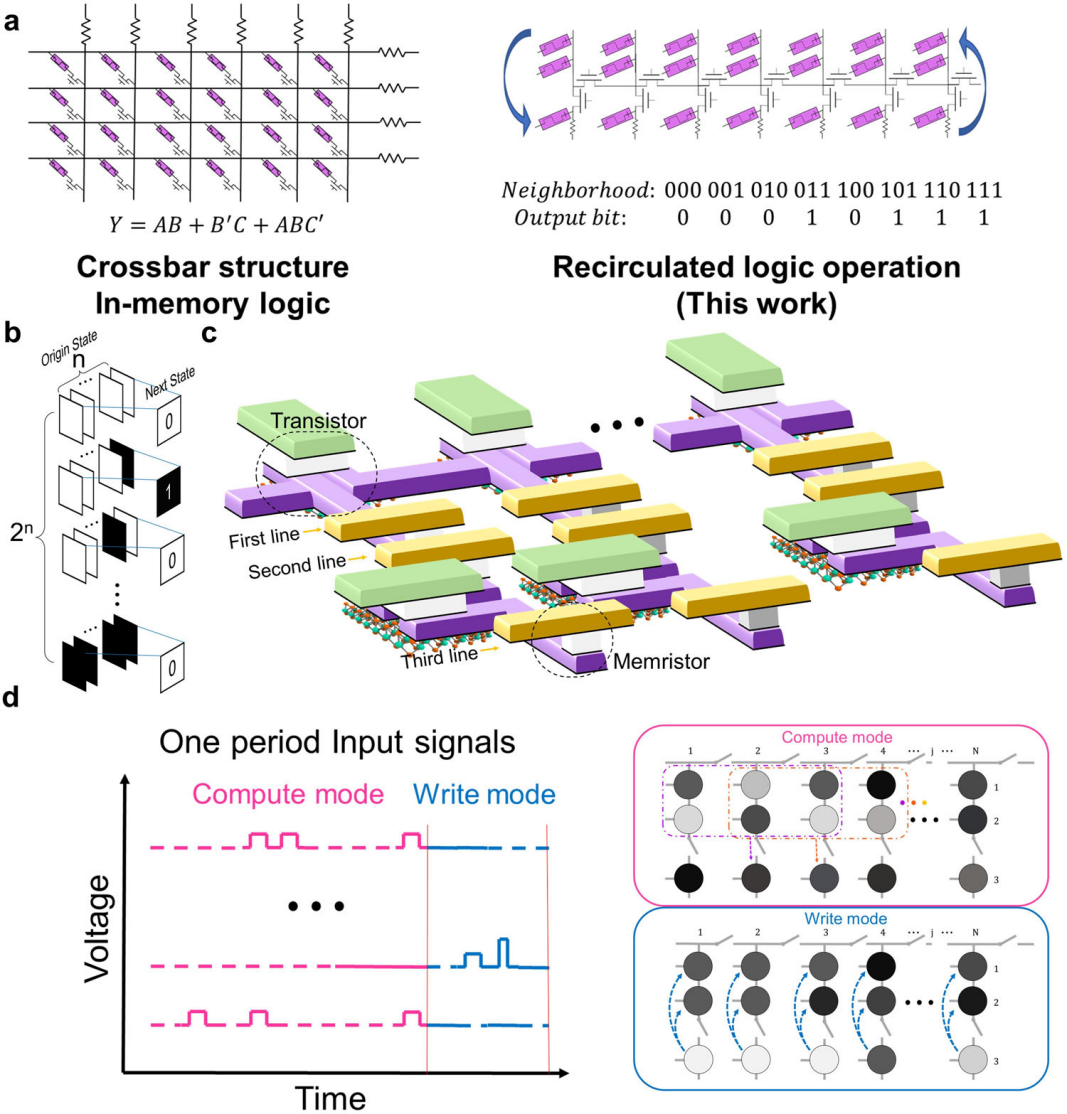
Overview of the RLOS. **a** The comparison of different computing units. The left panel depicts the crossbar structure; The right panel depicts the RLOS. **b** The schematic of 1D CA. **c** Implementation of RLOS by using the memristive array. **d** Schematic illustration of RLOS. The left panel is the schematic of input signals, which can be generated by the corresponding logic expression. In compute mode, the memristors are executed the logic operation to calculate the next state of CA. In write mode, the calculation result will be stored back into the original memristors. The right panel is the schematic of the circuit. The arrows indicate the direction of memristor evolution.

The schematic of the RLOS is built and shown in Fig. 1. The left panel of Fig. 1a depicts the crossbar structure based on memristors, primarily implementing in-memory logic. The right panel of Fig. 1a shows our proposed structure for RLOS, which is compatible with CA transition rules via multiplexing. The difference between the two circuits is in Supplementary Fig. 2 and Supplementary Note 1. In the crossbar structure, input and output memristors are always in the same row or column, resulting in larger hardware and power consumption for CA implementations. The corresponding CA crossbar structure implementation circuit in Supplementary Fig. 3 requires more hardware cost than RLOS. The comparison between the crossbar structure and RLOS for CA implementation is in Supplementary Table 1. Traditional implementation methods have redundancy due to inconsistent calculation steps required by different CA transition rules. RLOS perfectly matches CA transition rules and accommodates various rule lengths. Figure 1b shows an example of the basic form of the CA transition rule, with the next state of each cell determined by neighboring cell states. The state and inverse state of CA can be set as corresponding resistance states in the first and second lines of the memristive circuit (Fig. 1c), respectively. Transistors in the circuit prevent crosstalk during cell computations. Figure 1d is a schematic illustration of RLOS, with the left panel showing the input signal generated by the Boolean logic formula corresponding to the CA transition rule, and with the right panel showing the mapping relationship of the CA transition rule, which we will discuss later. Input signals are divided into two parts, representing compute and write modes. In compute mode, the circuit mainly performs logic operations according to corresponding transition rules. Since memristive devices use storage and calculation characteristics, there is no need for a read mode, and states can be calculated and stored directly in the device. In write mode, the calculation result is stored in the first two lines of the memristive circuit. Following this process, CA based on RLOS can be run step by step.

## Results

### Basic logic circuit

The circuits of RLOS are composed of memristors, transistors, and resistors. The basic step of the scheme can be decomposed into logic operation and state storage. First, we verify the basic NAND and AND logic operations.

For hardware implementation, the circuit needs to conform to certain parameter indicators. To ensure the feasibility of RLOS, the memristor should exhibit an On/Off ratio above 10^5^, which ensures that logic operations can still be completed in a memristor-transistor hybrid circuit. Therefore, conductive bridge random access memory (CBRAM) and MoS_2_-based transistors have been fabricated and measured. Conventional CMOS can only set the transistor on the bottom layer due to the doping process. The 2D material can be transferred to any substrate and maintain its functionality. As a result, our fabrication methods require less processes.

**Fig. 2:**
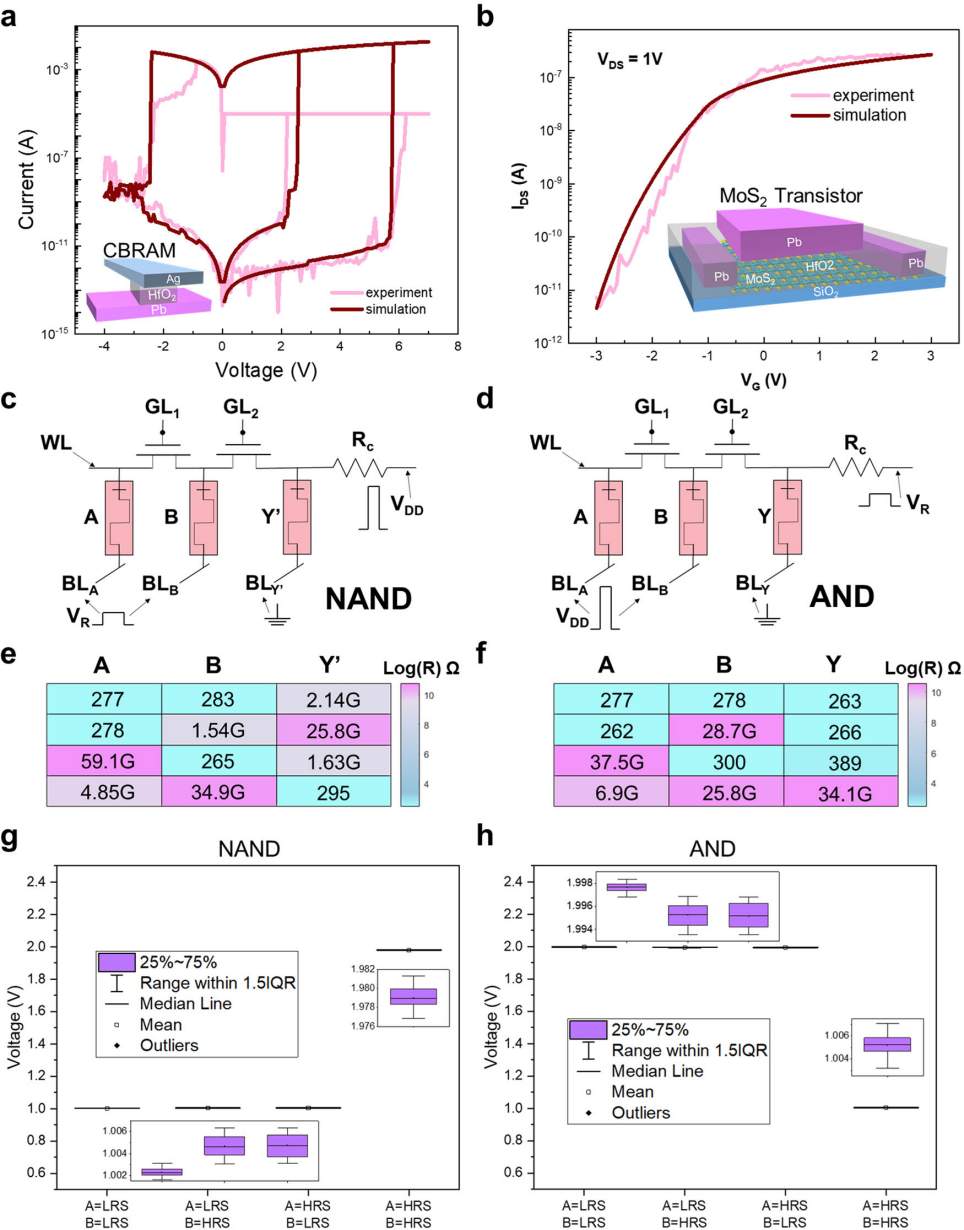
In-memory computing properties of the memristor and 2D transistor hybrid circuit. **a** The experimental and simulated *I–V* curve of the memristor. The inset image is a schematic of the structure for the memristor. **b** The experimental and simulated *I–V* curve of the transistor. The inset image is a schematic of the structure for transistor. **c** Basic circuit of NAND and **d** AND logic operations. Where *V*_dd_ = 2 V, *V*_R_ = 1 V and the pulse width is 40 ms. **e** Measured truth table of NAND and **f** AND logic operations. The states of the input A and B (output *Y*, output *Y*’) before (after) logic operation is read out as resistance shown by heatmaps. LRS and HRS are defined as logical “0” and logical “1”, respectively. **g** The deviation of voltage divided on the *Y*’ memristor under the applied *V*_dd_ and *V*_R_ with 100 times simulation in NAND circuit. **h** The deviation of voltage divided on the *Y* memristor under the applied *V*_dd_ and *V*_R_ with 100 times simulation in AND circuit.

The fabricated CBRAM has a top-to-down Ag/HfO_2_/Pt sandwiched structure (see inset of Fig. 2a). The transistor structure is shown in the inset of Fig. 2b. The 2D material MoS_2_ layer has been selected as channel material due to its moderate bandgap and high On/Off current ratio. The source, drain, and gate electrodes are made of Pt. Fabrication details can be found in Methods, and the schematic of the manufacturing process is in Supplementary Fig. 4. The current versus voltage (*I–V*) curve of the memristor is depicted in Fig. 2a, showing an On/Off resistance ratio >10^5^, which is sufficient for circuit operation. Specific experimental data can be found in Supplementary Fig. 5, and device-to-device variation analysis in Supplementary Fig. 6. The corresponding simulation curve is calculated by our theoretical model^29^, which can be used in the following verification. Detailed model results are in Supplementary Fig. 7. Figure 2b shows the gate voltage (*V*_*G*_) versus drain-to-source current (*I*_*DS*_) curve for a constant drain-to-source voltage (*V*_*DS*_) of 1 V, displaying a >10^5^ On/Off current ratio. The experimental data have been modeled using the α-power model^30^ (see Methods). Additional experimental results can be found in Supplementary Fig. 8. The high On/Off current ratio prevents crosstalk between different CA, which will be discussed thoroughly in the following section.

Figure 2c and d display memristor-based NAND and AND logic gates, respectively. These serve as the basic components of circuits implementing the RLOS. In these circuits, the resistance of the memristor represents the input and output signals, with *A* and *B* as input signals and *Y’* and *Y* as output signals. In the NAND logic gate, ${Y{{\hbox{'}}}}=\overline{{AB}}$ and ${Y{{\hbox{'}}}}=\overline{{AB}}X$ can be realized, where $X$ is the initial state of $Y\hbox{'}$ . Similarly, in the AND logic gate, both $Y={AB}$ and $Y={ABX}$ can be realized, where $X$ is the initial state of $Y$. To perform NAND and AND logic operations, trigger pulse signals *V*_*dd*_ and *V*_*R*_ are applied to the corresponding nodes to stimulate resistance state interaction between devices. Figure 2e, f show that the heatmap of the states of the input devices before operations and output devices after operations are read out as resistance values.

Note that the distribution of transistors and memristors in the logic gates differs from previous studies^31, 32^. The circuit comparison can be found in Supplementary Fig. 9. We have designed each logic operation using a small transistor–memristor circuit with broken symmetry (the original design has no transistors), which is crucial for preventing crosstalk in further RLOS implementations. Although the set of transistors affects the voltage division of the memristor in the circuit, the voltage reaching the memristor is still sufficient to turn it on, meaning that the Y memristor can operate normally. Moreover, the selector of R_c_ in Fig. 2c, d is sensitive to the variability of the memristor and the transistor. By employing Kirchhoff’s current law and Ohm’s law, the divider voltage between devices can be calculated. The resistance of R_c_ in our simulation is 1.05 × 10^8^ Ω (Following our calculations, we have established that R_c_ possesses a selectable range, specifically spanning from 6.04 × 10^7^ Ω to 1.5 × 10^8^ Ω). When the high-resistance state of the memristor is not high enough, we cannot even obtain a proper R_c_ to ensure the circuit’s functionality. Coupled with the limited conditions according to the logic rules, the value of R_c_ can be determined. The determined selection method can be found in Supplementary Fig. 10 and Supplementary Note 2. Figure 2g, h shows the divided voltage on the *Y* and *Y*’ memristor under 100 times spice simulation, respectively. It can be seen that our circuit exhibits good robustness, ensuring correct operation. The deviation of divided voltage on A and B memristors can be found in Supplementary Fig. 11a–d. The absolute values of divided voltage on A and B memristors are all less than 1 V, meaning the operation on the circuit cannot change the resistance states of memristors A and B. Supplementary Figs. 11e and 11f show the simulation results of the corresponding circuit. The “0” signal in the red dotted box has a significant difference in amplitude. Previous literature shows that the current of the “0” signals have almost the same amplitude. The reason for the difference in amplitude of the “0” states is the circuit asymmetry introduced by the transistors. The resistance of transistors is about 10^6 ^ Ω, while the low resistance of memristors is about 300 Ω. The voltage division of memristor A and the transistor is equivalent to that of memristor B, but the transistor has a larger resistance. Therefore, the current flowing through memristor A will be significantly smaller than the current flowing through memristor B.

### Cellular automata imbedded memristor-based in-memory computing scheme

**Fig. 3:**
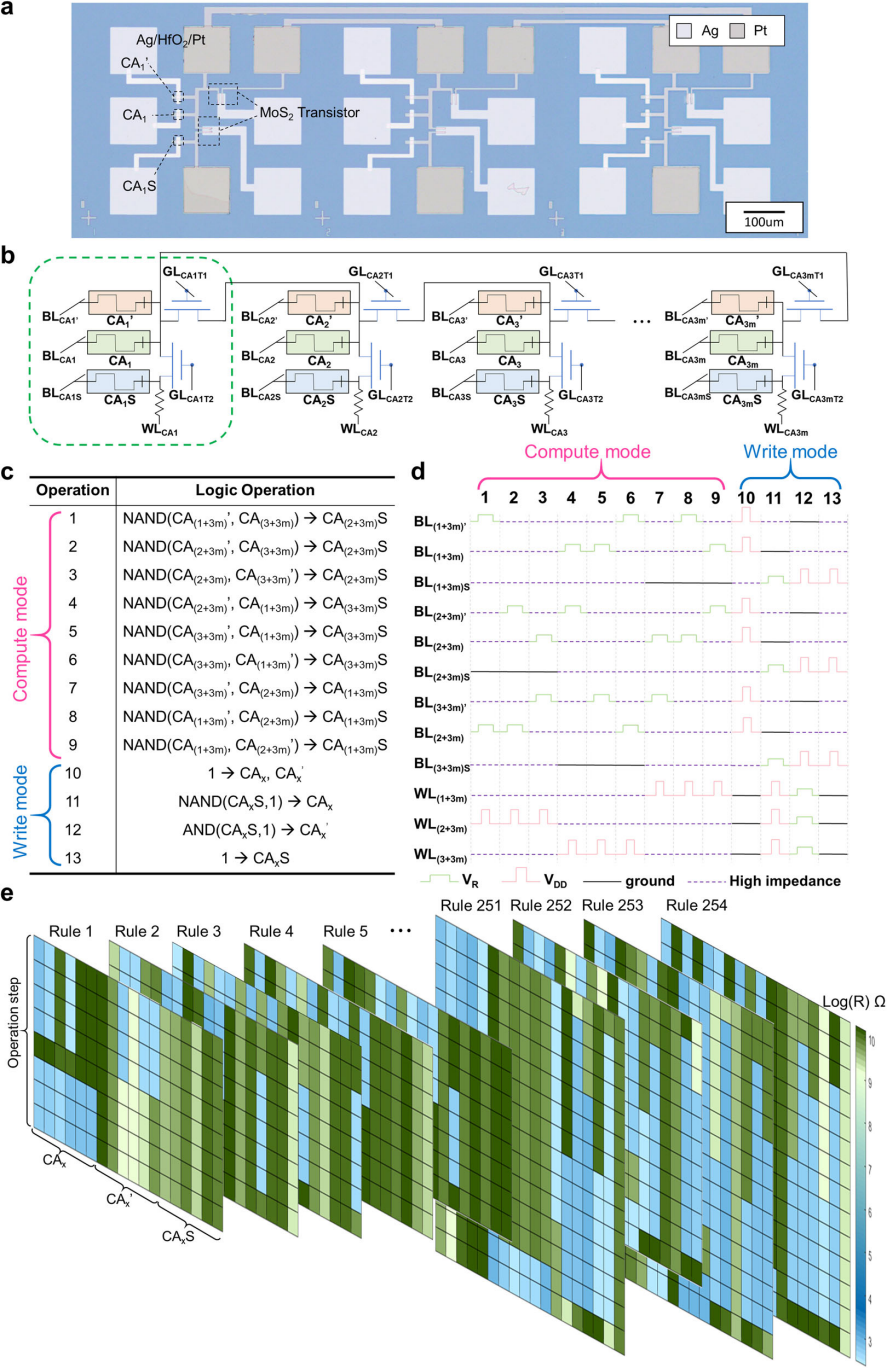
Illustration of RLOS-based 1D CA and demo of the ECA. **a** The microscope image of 1D CA circuit. **b** The circuit of 1D CA. The sub-circuits in green box represents one basic cell. The resistance in memristor CA_x_ represents the value of the CA. The resistance in memristor CA_x_’ represents the inverse value of the CA. The memristor CA_x_S is the auxiliary memristor whose initial state should be high-resistance state. **c** The logic operation of ECA rule 110. **d** The time sequence of the applied trigger signals to achieve the logic operation of ECA rule 110. **e** Memristors evolution diagram for entire rule of 1D ECA.

After analyzing the characteristic of devices and basic logic operation, we verify the circuit of RLOS. In this section, 1D CA with only two possible states per cell ($S=\{{{{{\mathrm{0,1}}}}}\}$) has been discussed. Thus, the evolution of the cell state could be described as transition rule $f:{\left\{{{{{\mathrm{0,1}}}}}\right\}}^{n}\to \{{{{{\mathrm{0,1}}}}}\}$ such that

1 $${s}_{i}(t+1)={f}_{1D}\left({s}_{i-r}\left(t\right),\,\ldots,\,{s}_{i}\left(t\right),\,\ldots,\,{s}_{i+r}\left(t\right)\right)$$

where $r$ (positive integer) is a parameter that represents 1D CA neighborhood, entailing to treat the neighborhood size as $n=2r+1$. The cell state is updated according to the CA transition rule. The 1D ECA have been defined with $r=1$, which results in a total of 256 rules. Among them, rule 0 and rule 255 change the state of all cells to 0 or 1, respectively. In terms of the device, this means that the devices are either all in a high-resistance state or all in a low-resistance state, which is easily achievable. Therefore, we will only discuss hardware realization for rules 1–254. Figure 3a shows the optical microscope image of the RLOS circuit. The corresponding circuit is depicted in Fig. 3b. The portion of the circuit enclosed by the green dashed square can be approximately considered as one basic cell within the CA. The transistor serves to calculate each cell separately, enabling parallel operations. As a demonstration, the rule 110 CA is presented, which is equivalent to the general Turing machine^33^. The schematic of the CA transition rule and memristor evolution diagram for rule 110 CA can be found in Supplementary Fig. 12a.

Example with rule 110, it can be written as:

2 $${s}_{h}\left(t+1\right)=\frac{1}{2}{{{{{\rm{sgn}}}}}}\left(\left(1-{s}_{h-1}\left(t\right)\right)*{s}_{h+1}\left(t\right)+\left(1-{s}_{h}\left(t\right)\right)*{s}_{h+1}\left(t\right)+{s}_{h}\left(t\right)*\left(1-{s}_{h+1}\left(t\right)\right)-0.5\right)+0.5$$

Where sgn(x) is signum function.

In fact, the CA transition rule can be treated as the Boolean function, which can be rewritten as Eq. (3) by using the Quine–McCluskey method^34^.

3 $${s}_{h}\left(t+1\right)=\overline{{s}_{h+1}(t)}{s}_{h+1}\left(t\right)+\overline{{s}_{h}\left(t\right)}{s}_{h+1}\left(t\right)+{s}_{h}\left(t\right)\overline{{s}_{h+1}(t)}$$

The above equation represents the corresponding operation in our RLOS. ${s}_{h}\left(t\right)$ represents the resistance state of the memristor at position $h$ after $t$ steps. Unlike traditional in-memory logic operations, the CA transition rule requires that all cells execute the above logic operations simultaneously. This is another reason why we should design RLOS to adapt to CA. Figure 3c is the table of logic operations for ECA rule 110, which can be derived from Eq. (3). Equation (3) can be converted to Eq. (4):

4 $${s}_{h}(t+1)=\overline{\overline{\overline{{s}_{h+1}(t)}{s}_{h+1}(t)}\cdot \overline{\overline{{s}_{h}(t)}{s}_{h+1}(t)}\cdot \overline{{s}_{h}(t)\overline{{s}_{h+1}(t)}}}$$

For the basic logic circuit (Fig. 2c, d), which can only implement AND and NAND logic, we have to convert the Boolean function to adapt to the corresponding format like Eq. (4). In circuit of RLOS (Fig. 3b), the resistance state of memristor number CA_x_^’^ corresponds to the $\overline{{s}_{x}\left(t\right)}$ in the Eq. (4). The resistance state of memristor number CA_x_ corresponds to the ${s}_{h}\left(t\right)$ in the Eq. (4). The resistance state of memristor number CA_x_S can be finally change to $\overline{{s}_{x}\left(t+1\right)}$ after the one step calculation. But initially, we use $X$ to represent the resistance state of memristor number CA_x_S. We need three operation steps to convert $X$ to $\overline{{s}_{x}\left(t+1\right)}$.

In the first operation step, we implement the operation: ${X}^{(1)}=\overline{{s}_{h}\left(t\right)\overline{{s}_{h+1}(t)}}$. In the second operation step, we implement the operation: ${X}^{(2)}=\overline{\overline{{s}_{h}\left(t\right)}{s}_{h+1}\left(t\right)}{X}^{(1)}$. In the third operation step, we implement the operation: ${X}^{(3)}=\overline{\overline{{s}_{h+1}(t)}{s}_{h+1}\left(t\right)}{X}^{(2)}$. Obviously, we can get the formula: ${X}^{(3)}=\overline{{s}_{x}\left(t+1\right)}$, which means that we divided the complex logic operation like Eq. (4) to three basic logic operations. The three basic logic operations can be implemented as described in Fig. 2c, d. The corresponding voltage signals can also be set in time sequence. However, we just analyze one logic operation. In CA transition rules, every cell should follow the transition rule. Therefore, the logic operation should be periodically executed, which can be found in operation steps 1–9 in Fig. 3c. Operation step 10 resets the first-line and second-line memristors. Operation steps 11 and 12 store the computed results in the first two lines memristors (first line: inverse value of CA, second line: value of CA). Operation step 13 resets the third-line memristor. This is a whole operation process of one period for CA. The memristor operation corresponding to each operation step is shown in detail in Supplementary Fig. 13.

The corresponding input voltage signal is shown in Fig. 3d. Figure 3e displays the memristor state evolution under different transition rules. A detailed illustration of the heatmap can be found in Supplementary Fig. 14. For three-cell CA (*r* = 1), a total of 254 ECA logic formulas will be discussed. The corresponding logic expressions can be found in Supplementary Data 1. These logic expressions can generate the respective input voltage signals. Supplementary Fig. 12b depicts the resistance state of each memristor at each operation step, which verifies the correctness of the RLOS. The complete set of 254 ECA corresponding memristor state evolution diagrams can be found in Supplementary Figs. 15 and  16. Therefore, we have verified the entire rules of 1D ECA.

### Majority classification algorithm

**Fig. 4:**
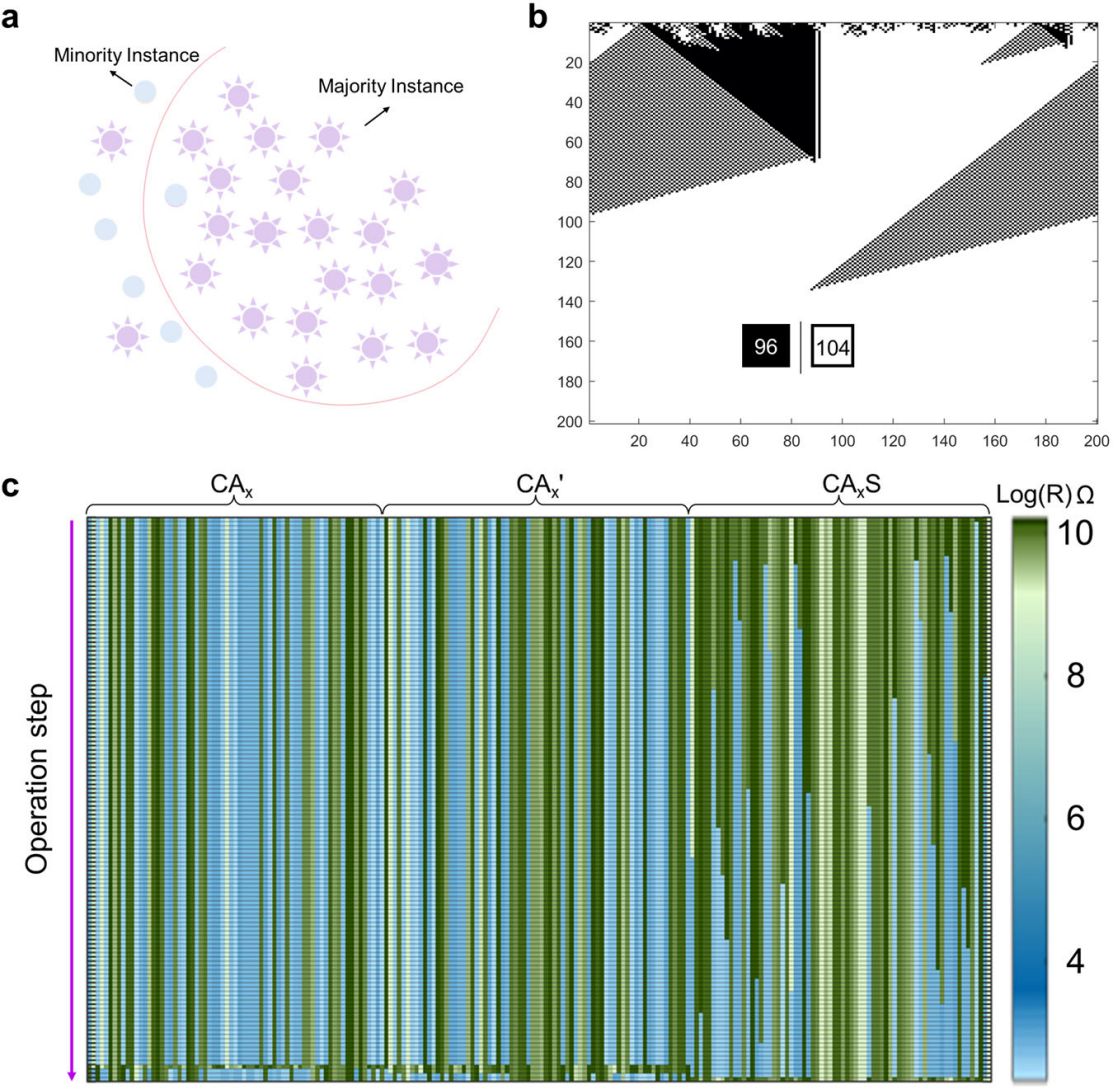
RLOS Implementation of majority classification. **a** The schematic of majority classification algorithm by CA. **b** The CA evolution diagram of majority classification. **c** The resistance state of memristor evolution in the designed circuit when running the majority classification algorithm.

The aforementioned ECA are of the ‘$r=1$’ type 1D CA. In fact, the RLOS can realize ‘$r={{{{\mathrm{1,2,3}}}}}$’ type 1D CA with the same circuit. Mitchell et al. proposed that 1D CA with three neighborhoods ($r=3,{n}=7$) can complete the majority classification task^35^. The specific description of the task is that there is a 0/1 sequence, in which 0 is the output if the number of 0 is dominant, and 1 is the output otherwise. Figure 4a shows the schematic of the majority classification task. The CA transition rules proposed by Mitchell et al. can complete the majority classification task. The rule selected by us is 0504058705000f77037755837bffb77f (hexadecimal)^35^. For CA, the random 0/1 sequence of the majority classification task can be regarded as the initial input value of the cell, and then the transition rules of the CA can be continuously run, and eventually, all states of the cell become 0 or 1, which is the classification result. Figure 4b shows the majority classification algorithm based on CA with 96 ones and 104 zeros as initial input. Clearly, the CA should transition to a 0 state in the end. This majority classification CA has been selected to execute via RLOS. Its transition rule can be converted to the corresponding Boolean function as A’B’EF’G + A’BCD’E + A’D’EG + BCDF + AC’DF + AC’EF + AB’CD’G + B’CDE’F’G’ + CDEF + ABC’E’G + ABC’EG’ + ABC’D + AC’DG + ABCD’E’G’ + CD’EG + ABDE + BCDG + ABF. The logic formula can be converted to 18 separate logic operations, as shown in Supplementary Table 2. The running period of this transition rule is 7, so the number of operations corresponding to one step is 131. Then, similar to the previous section, it can be converted to an operation list and generate the input signals. Figure 4c displays a diagram of the evolution of the memristor for the 70-input majority classification algorithm. To show the majority classification algorithms more clearly, we choose a 14-input data for classification (01001110100100). The final operation can prove the authenticity of the simulation. All states have been converted to the “0” state, as there are eight “0” states and six “1” states in the initial states. The complete state mapping of the memristor can be found in Supplementary Fig. 17, which verifies the correctness of our RLOS design.

CA, as a commonly used model, has many implementations. The most common implementation is coded by a personal computer. Due to the unique rules of CA and the Von Neumann architecture of computers, using personal computers to implement CA is extremely inefficient, and its computational complexity is O(n). A comparison of running time with a 3-cell CA as the baseline is displayed in Supplementary Fig. 18a, which demonstrates that our RLOS design has O(1) computational complexity. Another common CA implementation is programmed by FPGA. However, realizing CA based on FPGA has a shortcoming: different transition rules have different corresponding circuits. Supplementary Fig. 1a shows the flowchart of FPGA implementations. Encoder parts can be represented by different circuits under different CA transition rules. The encoder for running the CA-based majority classification algorithm can be presented as a circuit in Supplementary Fig. 19. It is easy to observe that RLOS has lower hardware costs (up to 79 times) than FPGA implementations, which are also depicted in Supplementary Fig. 18b.

### Edge detection algorithm

**Fig. 5:**
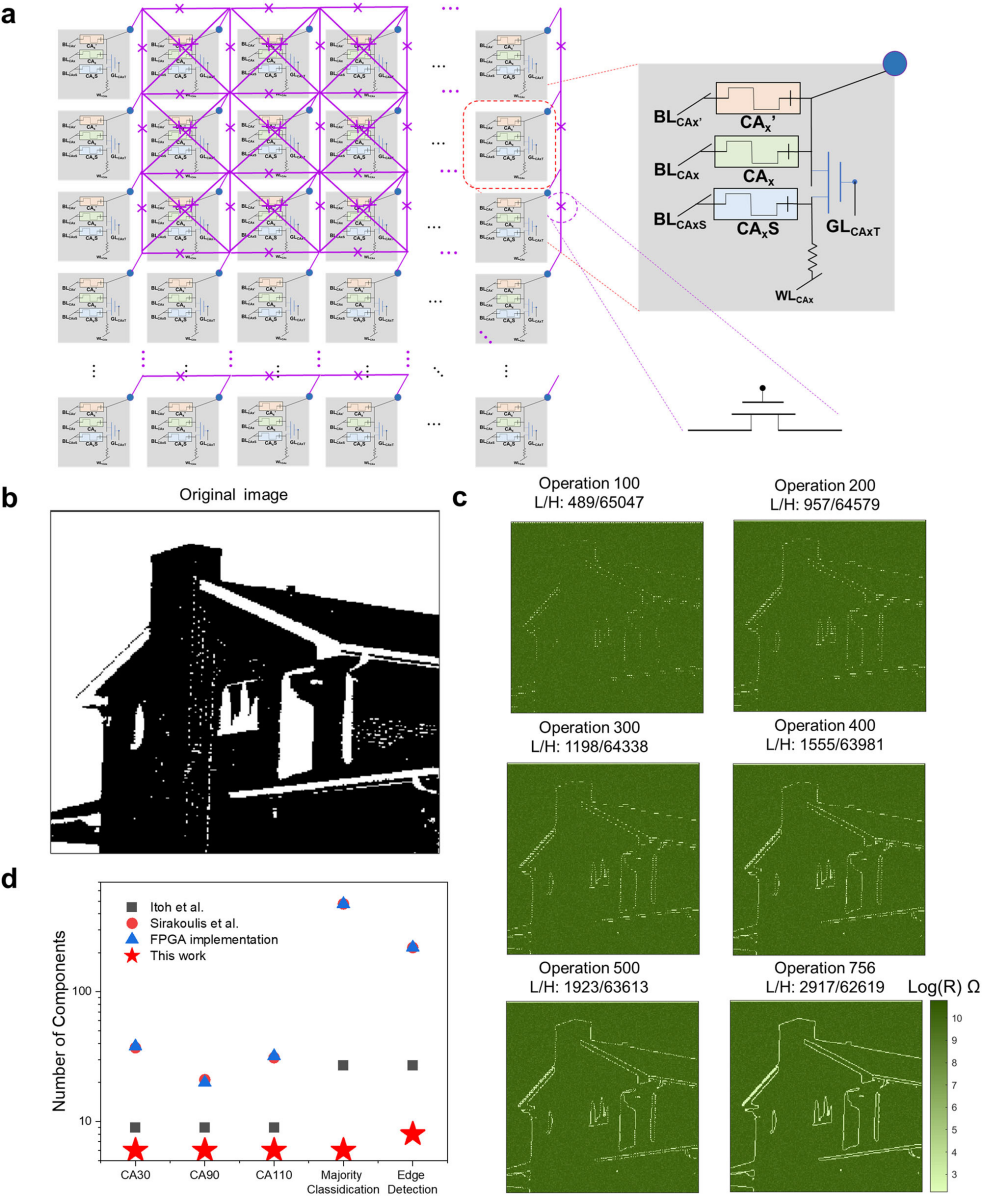
2D CA implementation based on RLOS. **a** The circuit of 2D CA. The gray box presents our basic unit of RLOS. The dash line presents the transistor. **b** Original 256 × 256 image (house) is from USC-SIPI image database. The origin image is RGB format. We use Matlab to convert it to black and white image. The baseline for black and white processing (commend: im2bw) is 0.54. **c** Simulated resistance state of memristor evolution when running the edge detection algorithm. (1) At the end of 100 operations, the ratio of the number of low-resistance states to the number of high-resistance states of the memristor (L/H) is 489/65047. (2) At the end of 200 operations, L/H is 957/64579. (3) At the end of 300 operations, L/H is 1198/64338. (4) At the end of 400 operations, L/H is 1555/63981. (5) At the end of 500 operations, L/H is 1923/63613. (6) At the end of 756 operations, L/H is 2917/62619. **d** The hardware cost comparison between RLOS and previous work.

To further explore RLOS, 2D CA have been introduced and discussed. Figure 5a presents the circuit of RLOS for implementing 2D CA. The basic components are displayed in the gray box. As mentioned in the previous section, the selector of R_c_ is sensitive. The boxes are interconnected by transistors to prevent crosstalk during various CA operations. In 2D CA, the next state of each cell is determined by the states of its eight neighboring cells and its own state. Consequently, each component interconnects with eight other components.

The edge detection algorithm was chosen to verify the RLOS-based 2D CA. Edge detection is an image processing technique used to find the boundaries of objects within images. In 2D CA, the transition rule can be defined as follows: In the 9 neighboring cells, (1) if the number of “1” state cells is equal to 6, 7, or 8, then the new state of the central cell will be black; (2) in any other case, the new state of the central cell will be white^36^. Similar to the previous section, this transition rule can be converted to Boolean logic, which can then generate the corresponding operation and input signal. The specific description of the transition rule can be found in Supplementary Note 3. The classic image in Fig. 5b has been selected as the input state. The selected image is from USC-SIPI dataset. The image has 256 × 256 pixels, meaning 65,536 input values. Figure 5c displays the state of the memristors under various operations. The memristor states under operation numbers 100, 200, 300, 400, 500, and 756 are depicted. Operation number 756 is the largest possible number based on the case of the edge detection rule. It can be observed that the number of low-resistance state memristors has gradually increased from 0 to 2917 (operating from high-resistance state to low-resistance state). After 756 operations, the outline of the image has been detected, which verifies the validity of RLOS in 2D CA. Figure 5d compares the hardware cost of the RLOS under different tasks. RLOS utilizes the in-memory computing characteristic of memristors, enabling significant hardware cost savings. It is worth noting that FPGA implementations may still have room for optimization with lower hardware costs. However, due to the different computing mechanisms, FPGA implementation cannot achieve lower hardware costs than our RLOS. Furthermore, the CMOS custom solution results in a specific circuit configuration, which cannot flexibly convert the different transition rules of the CA. Therefore, rule 90 and rule 150 have been selected for comparison^37^. The hardware cost of RLOS is more than two times lower than that of CMOS custom solutions. These comparisons confirm RLOS’s low hardware cost characteristics.

### Reservoir computing system based on cellular automata

**Fig. 6:**
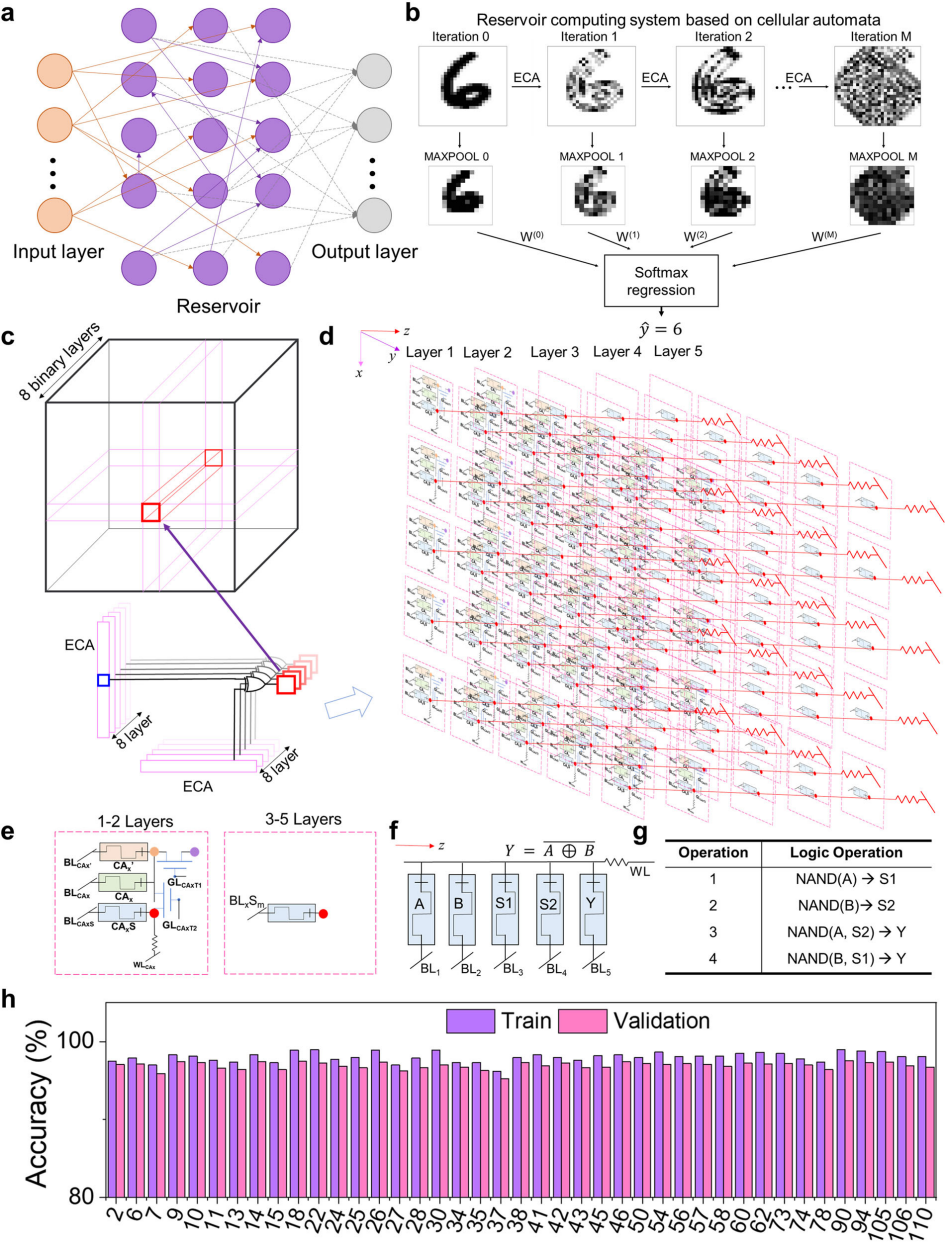
RLOS implementation of a reservoir computing system based on ReCA. **a** Schematic of reservoir computing scheme. **b** Scheme of the proposed CA-based classifier applied to a MNIST sample. **c** The schematic of CA operation in ReCA. **d** The schematic of the circuits for CA operation in ReCA**. e** The schematic of units in (**d**)**. f** Basic circuit of the required gate. **g** Cascading method of the required gate**. h** Performance of the ReCA based on RLOS using ten iterations of different ECA transition rules.

The proliferation of the Internet of Things has given rise to a new paradigm, edge computing (EC), which incorporates data processing at the edge of the network^38^. EC moves a portion of the storage and computes resources out of the data center and closer to the source of the data itself, reducing transfer latency and data movement. Machine learning (ML) is the most common algorithm that needs to be deployed on edge nodes. However, edge nodes typically have limited processing capabilities in terms of area and power. Therefore, it is crucial to develop new methodologies for implementing energy-efficient hardware. ML algorithms deployed on edge nodes can be realized based on CA^10,39–41^. Reservoir Computing is a ML alternative characterized by simplicity and a computationally inexpensive learning process, making it suitable for edge applications (Fig. 6a). In addition, a reservoir computing system based on cellular automata (ReCA) has been recently proposed^10^. The implementation of ReCA based on RLOS can significantly reduce hardware costs and data movement.

Figure 6b shows a schematic of the dynamic behavior of the ReCA classifier. ECA transition rules have been used to iterate the data, and Softmax regression has been employed to predict the final results. Our RLOS primarily realizes the ECA iteration process. The specific iterative process can be found in Fig. 6c. The training images are 2D grayscale images from the MNIST database, and each pixel of the images is characterized by an 8-bit signal value. Therefore, one input can be divided into 8 binary layers. We then iterate rows and columns independently using ECA transition rules with fixed boundary conditions and combine the resulting vectors using a bitwise XOR operation. This process can be implemented by our RLOS. The corresponding circuits are shown in Fig. 6d. We use a 3D memristive array to implement the process. Due to the in-memory logic characteristics, this design has lower data movement (Supplementary Fig. 20). The subcircuit units of each layer can be found in Fig. 6e. The first and second layers are similar to the ECA circuits in Fig. 3. Specific complete circuits of the first and second layers can be found in Supplementary Figs. 21 and  22, respectively.

The circuit of the first layer executes the row direction ECA iteration, and the circuit of the second layer executes the column direction ECA iteration. The bitwise XOR operation can be realized by the z-direction circuits. The circuit in the z-direction can be found in Fig. 6f, and the corresponding operation can be found in Fig. 6g. The circuits in the first and second layers have enough transistors, so the current crosstalk in z-direction circuits can be negligible. The resistance value of the memristors in the fifth layer represents the final result after calculation. Our design can significantly reduce hardware costs and data movement in this process. Figure 6h displays the recognition accuracy under different ECA transition rules. The recognition accuracies of the selected transition rule training results are all over 96%. Our scheme proposes a new hardware implementation that obtains lower hardware costs and lower data movement, which also showcases the potential of hardware implementation of edge computing based on RLOS.

## Discussion

In summary, we have proposed an RLOS to realize CA. A memristor array is used to store and compute the values of cellular automata. Our design demonstrates extremely low hardware costs. The data fed into our model have been validated by experiments. The entire rule of ECA, the majority classification algorithm, and the edge detection algorithm have been verified. The hardware cost has been compared to the conventional FPGA approach, showing a reduction of up to 79 times. Finally, we have proposed reservoir computing based on RLOS. This work introduces a design to realize CA, which can greatly contribute to the development of hardware equipment for edge computing.

## Methods

### Device fabrication

The designed 2T −3R structure was fabricated as follows: First, metal-organic chemical vapor deposited (MOCVD) MoS_2_ on sapphire substrate was transferred and pattered on the 300 nm SiO_2_/p+ Si substrate. CVD monolayer MoS_2_ film was grown on Si/SiO_2_ substrate, bought from Shenzhen SixCarbon Technology Co., Ltd. Then, after electron-beam lithography (EBL) and electron-beam evaporation (EVP) Ti/Pt with 2 nm/35 nm thickness was selected as the contact metal for MoS_2_ transistor and bottom electrode for memristor. After that, the 10 nm-thick HfO_2_ layer was deposited by atomic layer deposition with a temperature of 200 °C, which acts as both the gate dielectric of MoS_2_ transistor and the resistive switching layer of memristor. Next, after carrying EBL and EVP process for another two times, Ag with 40 nm as top electrode of memristor and Pt with 40 nm as gate metal of MoS_2_ transistor were deposited.

### Measurement set-up

The basic electrical behaviors of the memristor and transistor were characterized at room temperature in a probe station connecting to a semiconductor parameter analyzer (Agilent B1500).

The simulation of the devices was realized with a personal computer (PC). A Matlab program has been written to simulate the devices. A Monte Carlo model has been established to describe the behaviors of the memristor. The rates of particles were selected as probability weight to execute the Monte Carlo method. The rate function can be written as: ${r}_{x}={\nu }_{f}\cdot {{\exp }}\left(-\frac{E-\alpha q\Delta V}{{k}_{B}T}\right)$, where $E$ is the activation energy, $\alpha$ is a parameter related to $q\Delta V$, ${k}_{B}$ is Boltzmann constant, $T$ is temperature. The corresponding parameters can be fitted from experiments. The thorough memristor model could be found from our previous work^29^. The simulation of the transistor model has used the function: ${I}_{{DS}}={K}_{S}\frac{W}{L}{\left({V}_{G}-{V}_{T}\right)}^{\alpha }$.

## Supplementary information

Supplementary Information

Description of Additional Supplementary Files

Supplementary Data 1

## Data Availability

The data that support the findings of this study are available from the corresponding author upon reasonable request.
