# Supplementary material for: Cellular automata imbedded memristor-based recirculated logic in-memory computing
Source: Nat Commun. 2023 May 10;14:2695. doi: 10.1038/s41467-023-38299-7 (PMC10172358; doi:10.1038/s41467-023-38299-7)
Supplement: Supplementary file 1 — Supplementary Information [file 41467_2023_38299_MOESM1_ESM.pdf]

Supplementary information

Cellular automata imbedded memristor-based recirculated logic in-memory computing

Yanming Liu[1, 2, †], *He Tian*[1, 2, †, *], *Fan Wu*[1,2, †], *Anhan Liu*[1,2], *Yihao Li*[3], *Hao Sun*[1,2], *Mario Lanza*[4], *Tian-Ling Ren*[*1,2]

[1] School of Integrated Circuits, Tsinghua University, Beijing 100084, China.

[2] Beijing National Research Center for Information Science and Technology (BNRist), Tsinghua University, Beijing 100084, China.

[3]Weiyang College, Tsinghua University, Beijing 100084, China.

[4]Physical Science and Engineering Division, King Abdullah University of Science and Technology (KAUST), Thuwal, Saudi Arabia

†These authors contributed equally to this work.

Corresponding to: RenTL@tsinghua.edu.cn, tianhe88@tsinghua.edu.cn

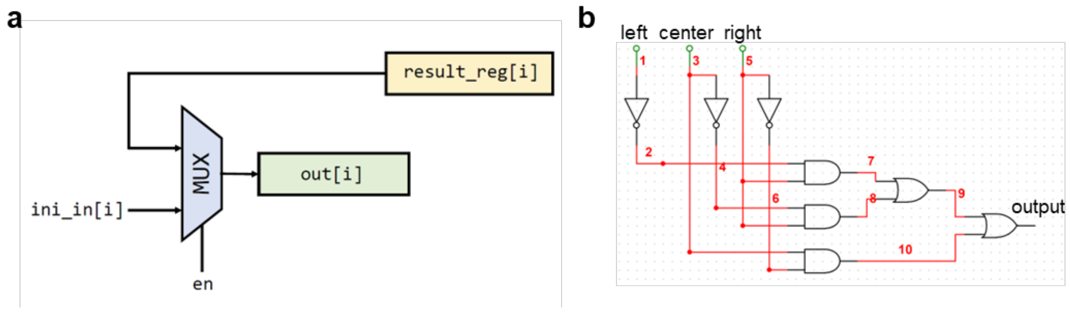

Supplementary Figure 1. Block diagram of the CA cell architecture implemented by FPGA. (a) FPGA implementation scheme of the CA. (b) Rule Switching Logic unit for 1-D elementary CA Rule 110.

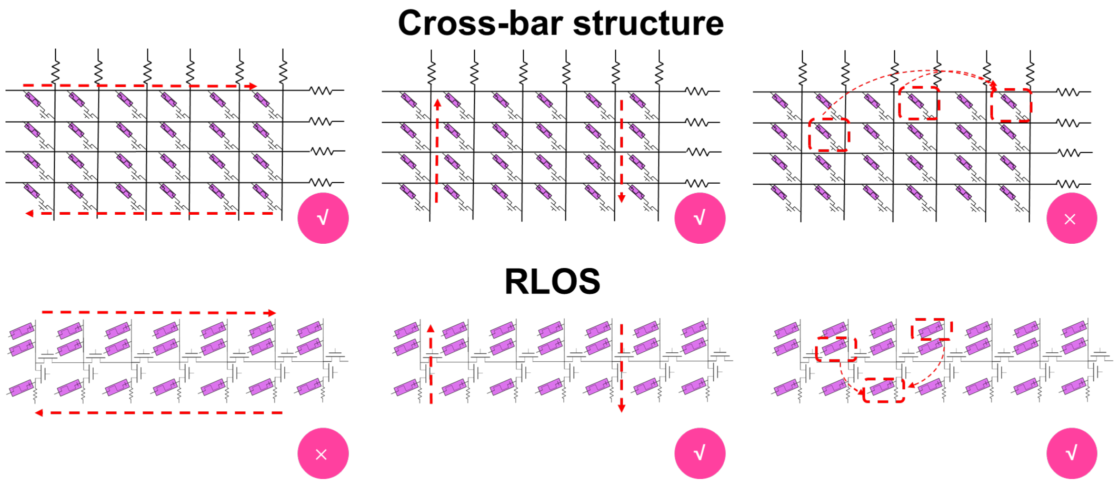

Supplementary Figure 2. The functional comparison between cross-bar structure and RLOS.

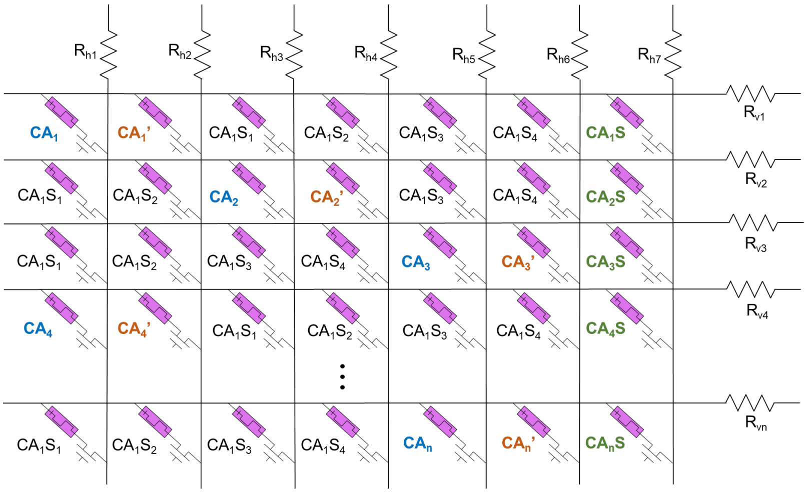

Supplementary Figure 3. The cellular automata implementation based on cross-bar structure.

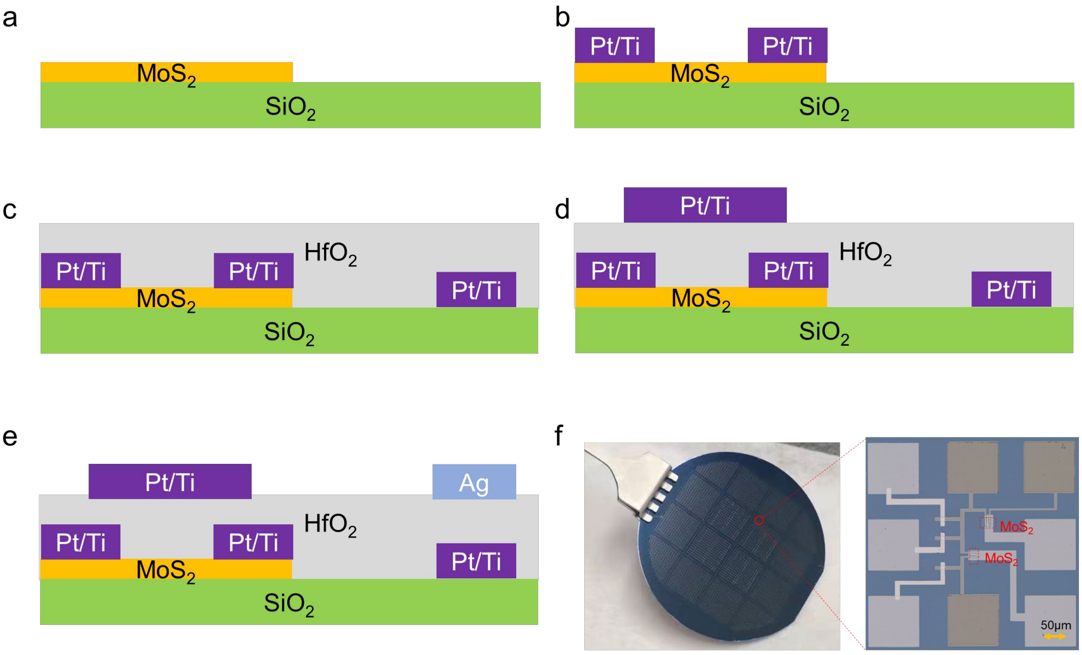

Supplementary Figure 4. RLOS devices manufacturing process. (a) Transfer MoS_2. (b) Using electron-beam lithography (EBL) and electron-beam evaporation (EVP) to fabricate Ti/Pt 2 nm/35 nm thickness electrode. (c) Deposited the 10 nm-thick HfO_2 layer by atomic layer deposition. (d). Carrying EBL and EVP process to fabricate Pt with 40 nm as gate metal of MoS_2 transistor (e) Carrying EBL and EVP process to fabricate Ag with 40 nm as top electrode of memristor. (f) The wafer level image and microscope image for 1 unit structure.

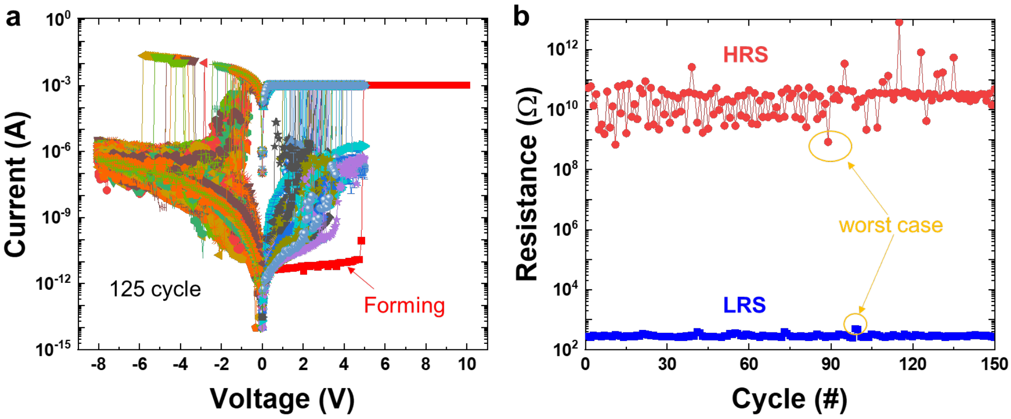

Supplementary Figure 5. The experimental data of memristor. (a) The 125 cycle of I-V curve of Ag/HfO_2/Pt memristors. (b) Measured resistance state result for 150 cycles.

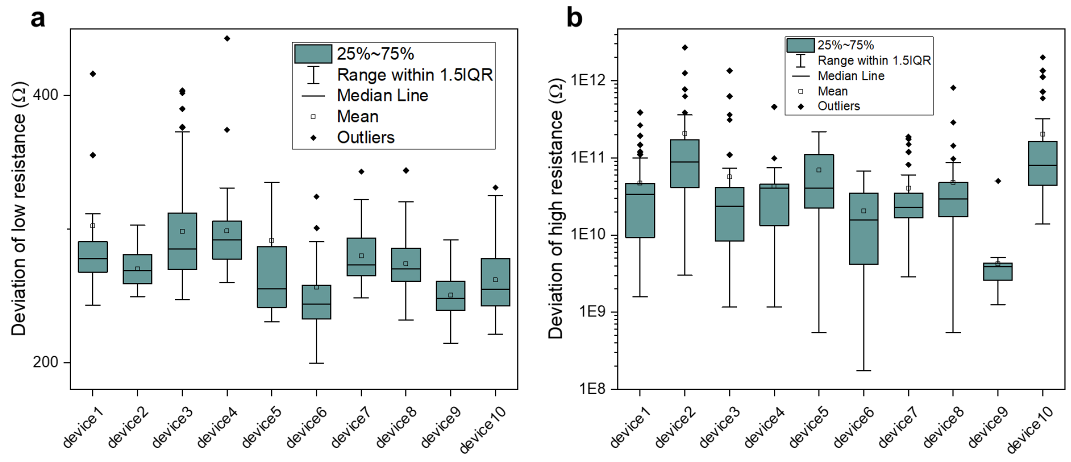

Supplementary Figure 6. The device-to-device variation of fabricated memristors. (a) The deviation of low resistance of memristor with 10 devices. (b) The deviation of high resistance of memristor with 10 devices.

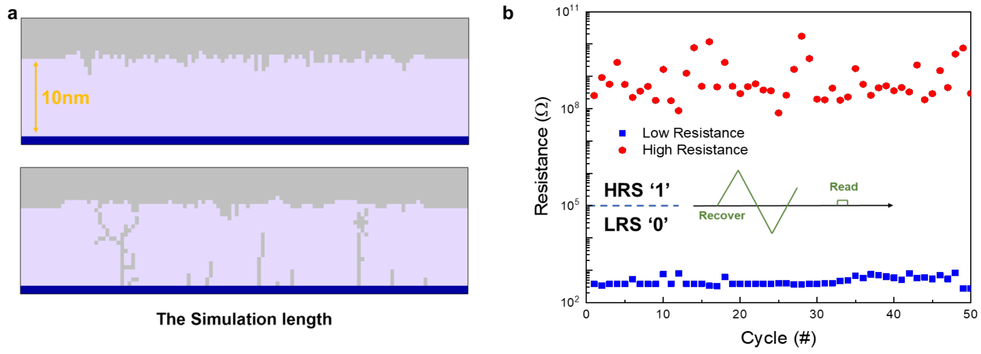

Supplementary Figure 7. Memristor Simulation Model. (a) Particle distribution of memristor. (b) Stability of 50 switching cycles under DC mode. The model can be found in Methods[1].

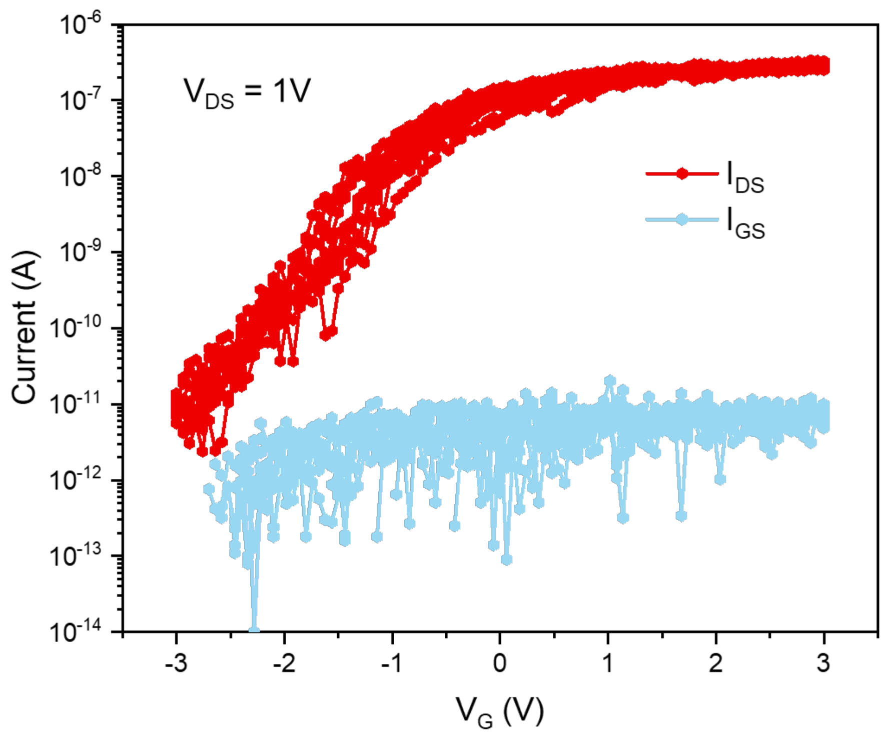

Supplementary Figure 8. The transfer curves of ten typical devices at $V_{DS} = 1$ V.

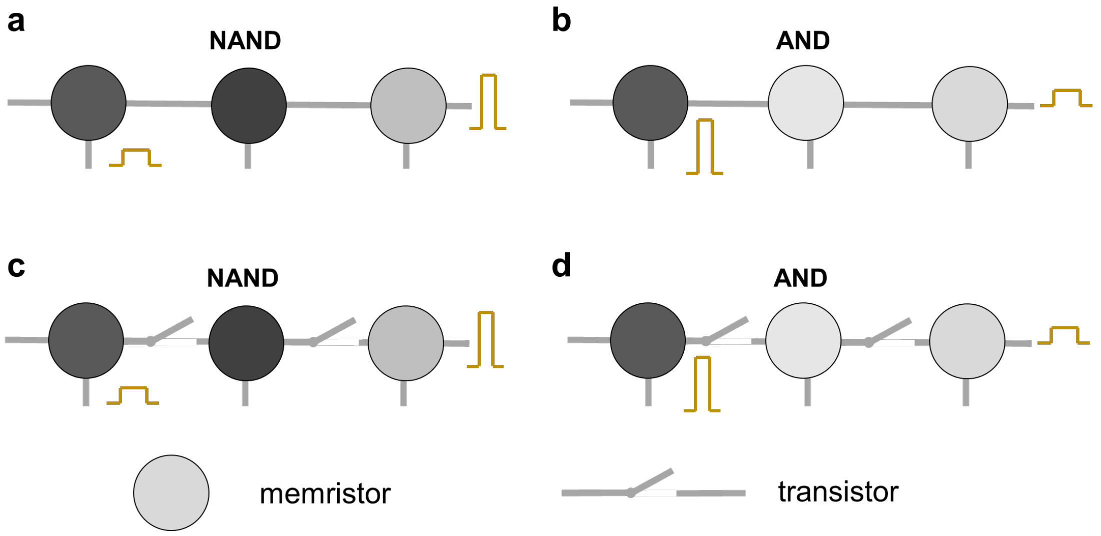

Supplementary Figure 9. The schematic of NAND and AND logic operations. (a) sub-circuit of NAND and (b) AND logic operations executed by memristor[2]. (c) sub-circuit of NAND and (d) AND logic operations executed by memristor, transistor hybrid circuit. The circle presents for memristor and the switch presents for transistor.

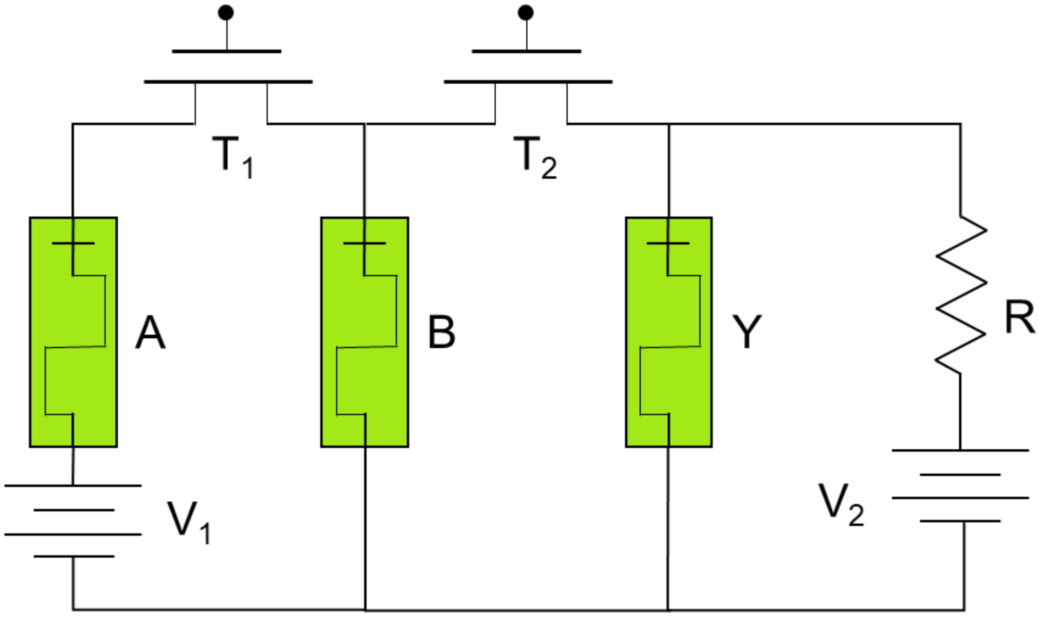

Supplementary Figure 10. The simplified circuit of the NAND RRAM-based circuit. The calculation of the circuit can be found in Supplementary Note 1.

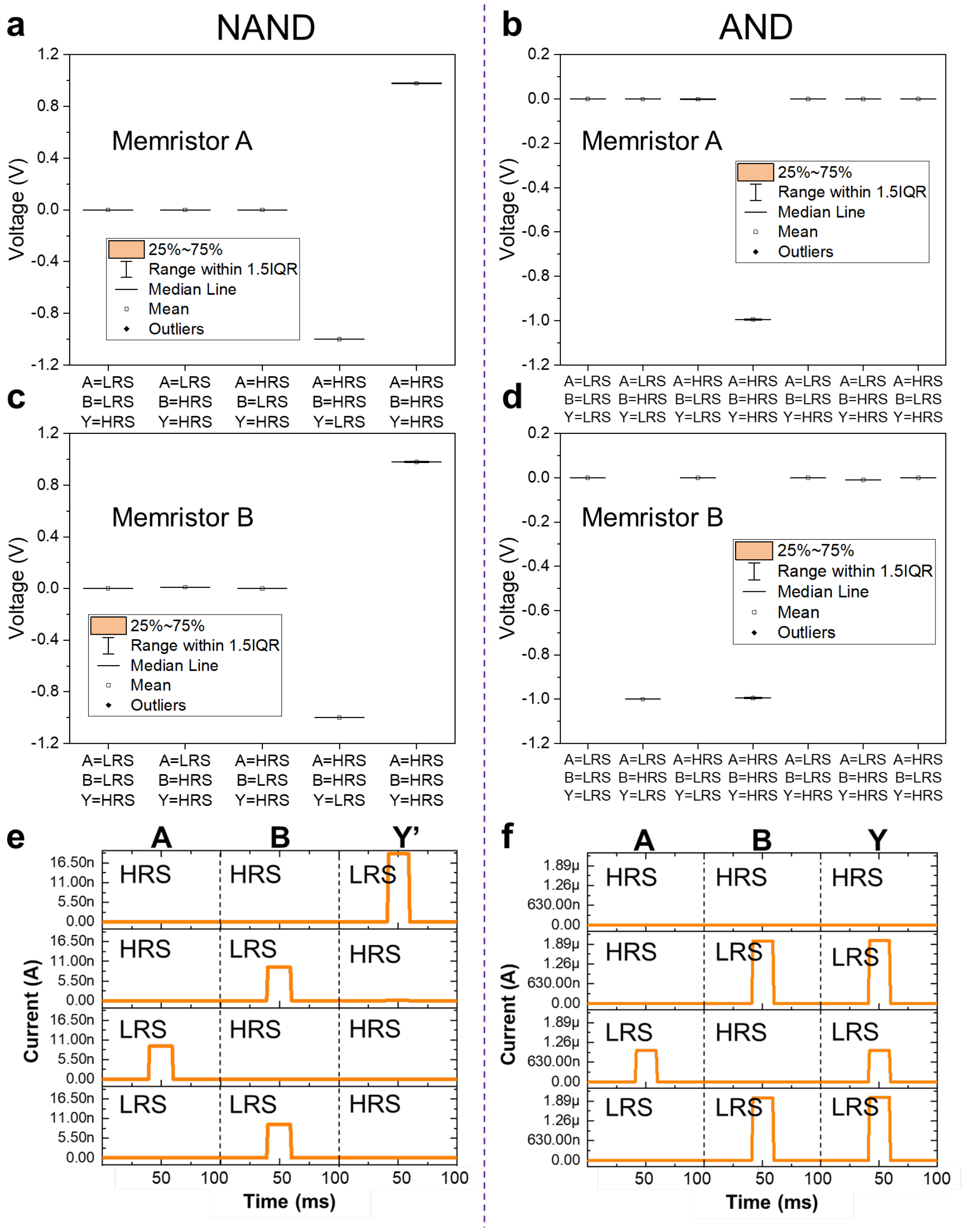

Supplementary Figure 11. a, The deviation of voltage divided on the A memristor under the applied V_{dd} and V_R with 100 times simulation in NAND circuit. **b**, The deviation of voltage divided on the A memristor under the applied V_{dd} and V_R with 100 times simulation in AND circuit. **c**, The deviation of voltage divided on the B memristor under the applied V_{dd} and V_R with 100 times simulation in NAND circuit. **d**, The deviation of voltage divided on the B memristor under the applied V_{dd} and V_R with 100 times simulation in AND circuit. **e,** Simulation results of states of output after NAND and **f,** AND logic operations versus the state combinations of inputs A and B.

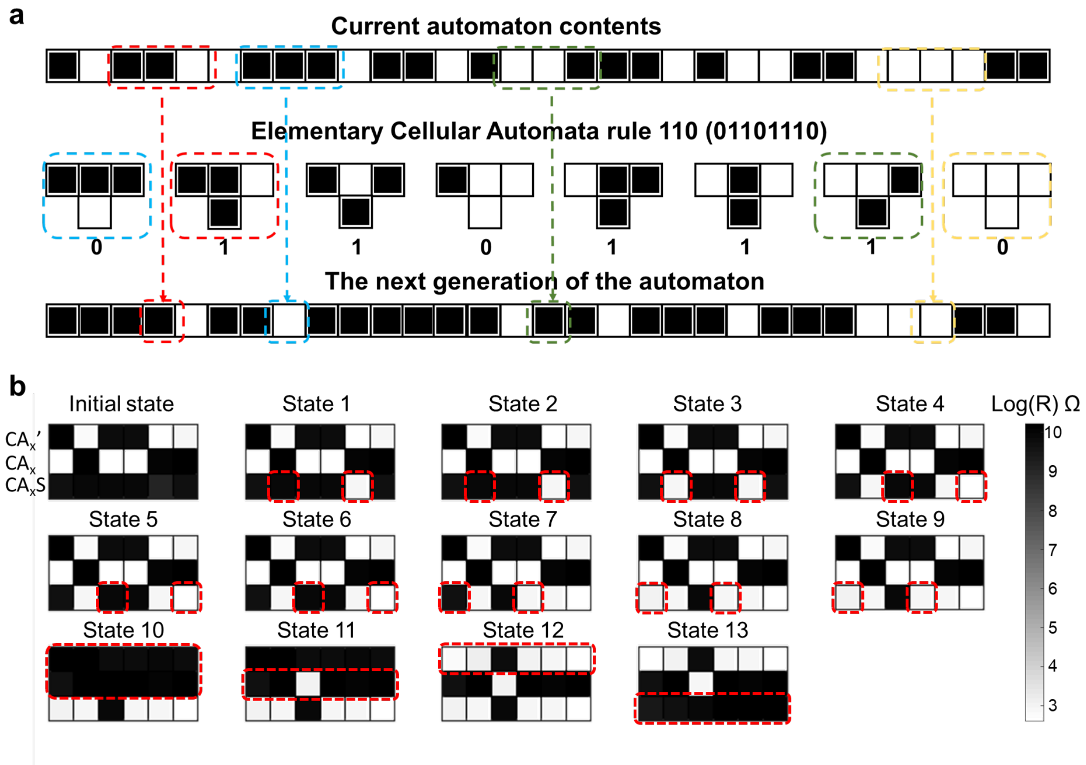

Supplementary Figure 12. Rule Switching Logic unit for Majority classification algorithm based on 1-D CA. (a) the transition rule of elementary cellular automata rule 110. (b) The evolution diagram of the memristor array corresponding the rule 110. The first line presents the inverse value of cellular automata. The second line presents the value of cellular automata. The third line presents the value of auxiliary memristor.

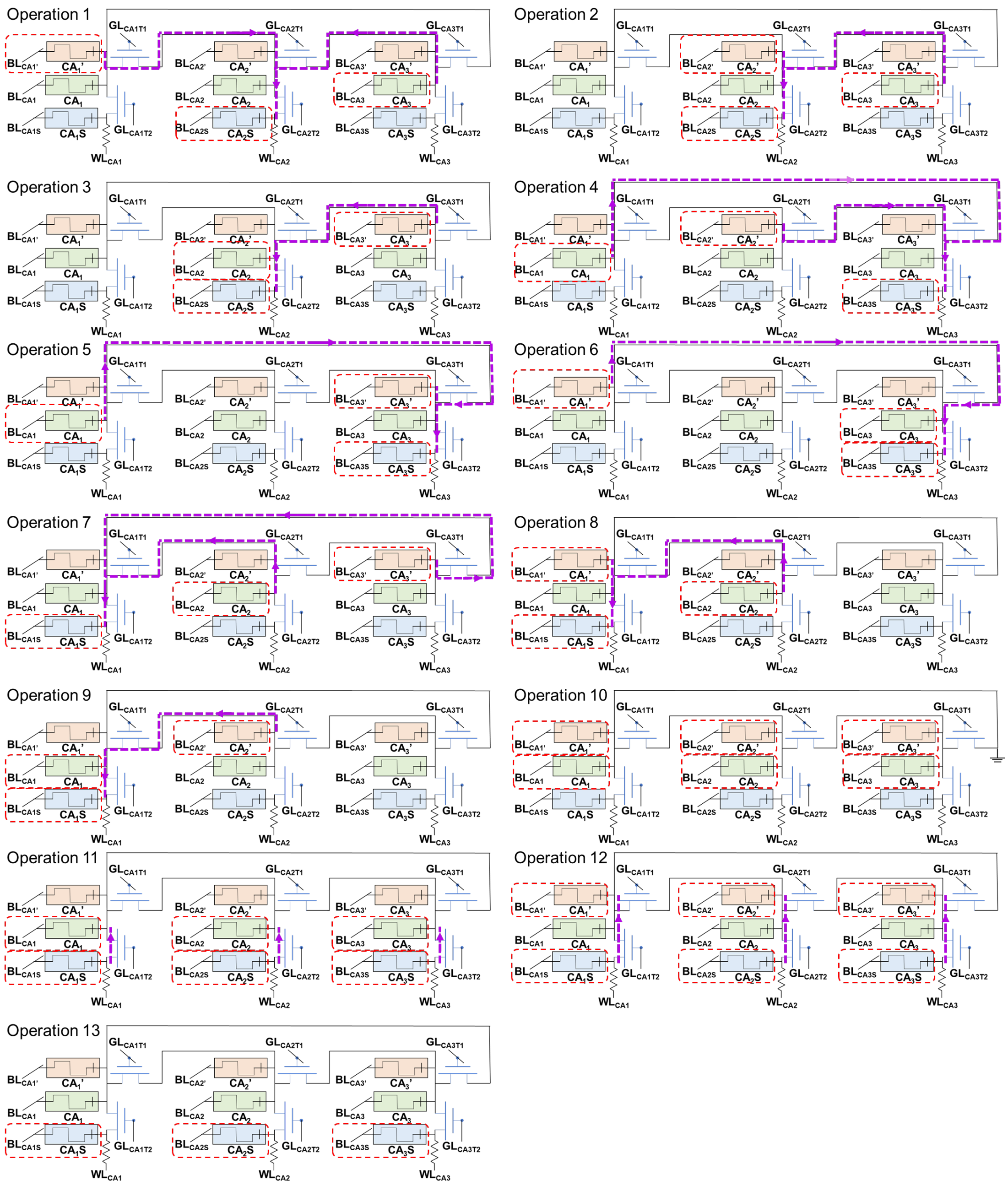

Supplementary Figure 13. The schematic of executed memristor and current direction under each operation.

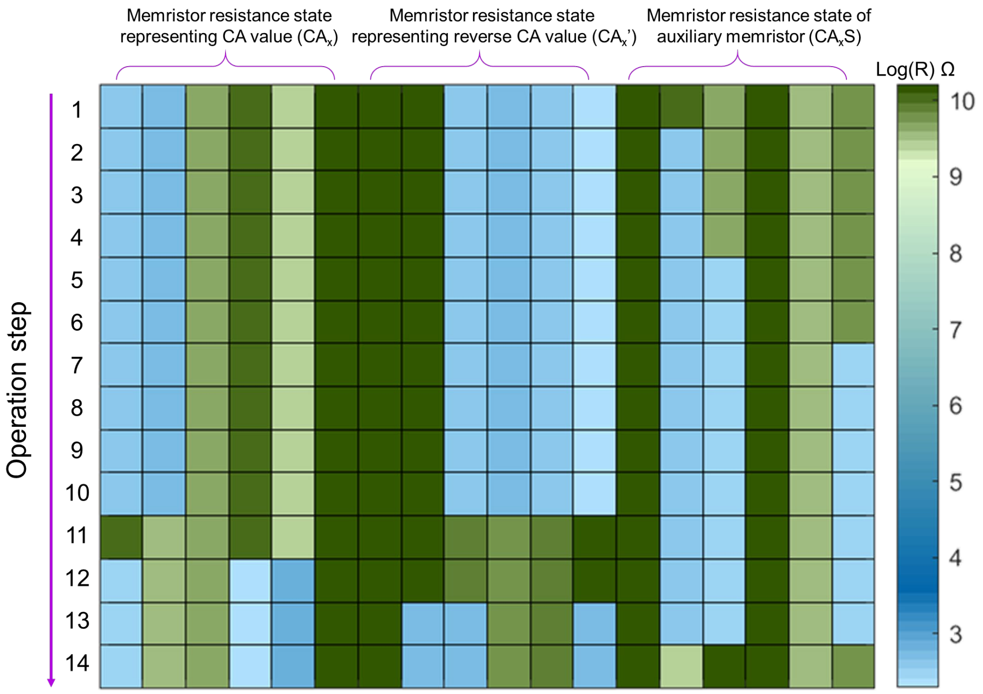

Supplementary Figure 14. The heatmap of evolution of memristor resistance under CA 110 transition rule. We selected 6 cellular to simulate the evolution of CA based on different transition rules. The first 6 columns are the evolution of memristor resistance state representing the corresponding CA values. The 7-12 columns are the evolution of memristor resistance state representing the corresponding reverse values of CA. The 13-18 columns are the evolution of resistance state of the auxiliary memristors. The output of one step of transition rule can be read from the last row of the heatmap. Therefore, the heatmaps can reflect the specific process of evolution.

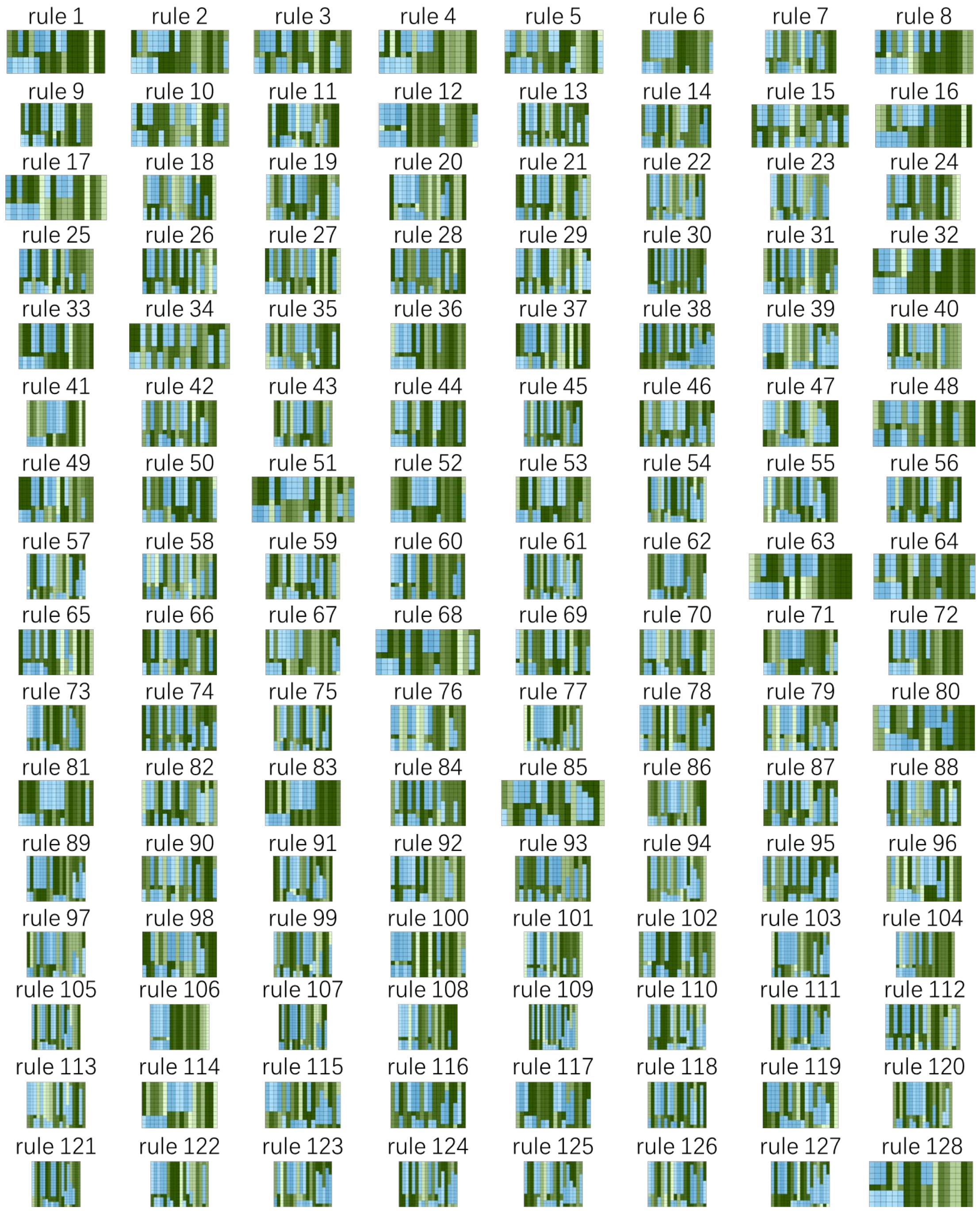

Supplementary Figure 15. Memristors evolution diagram for entire rule of 1D elementary cellular automata (Rule 1-128).

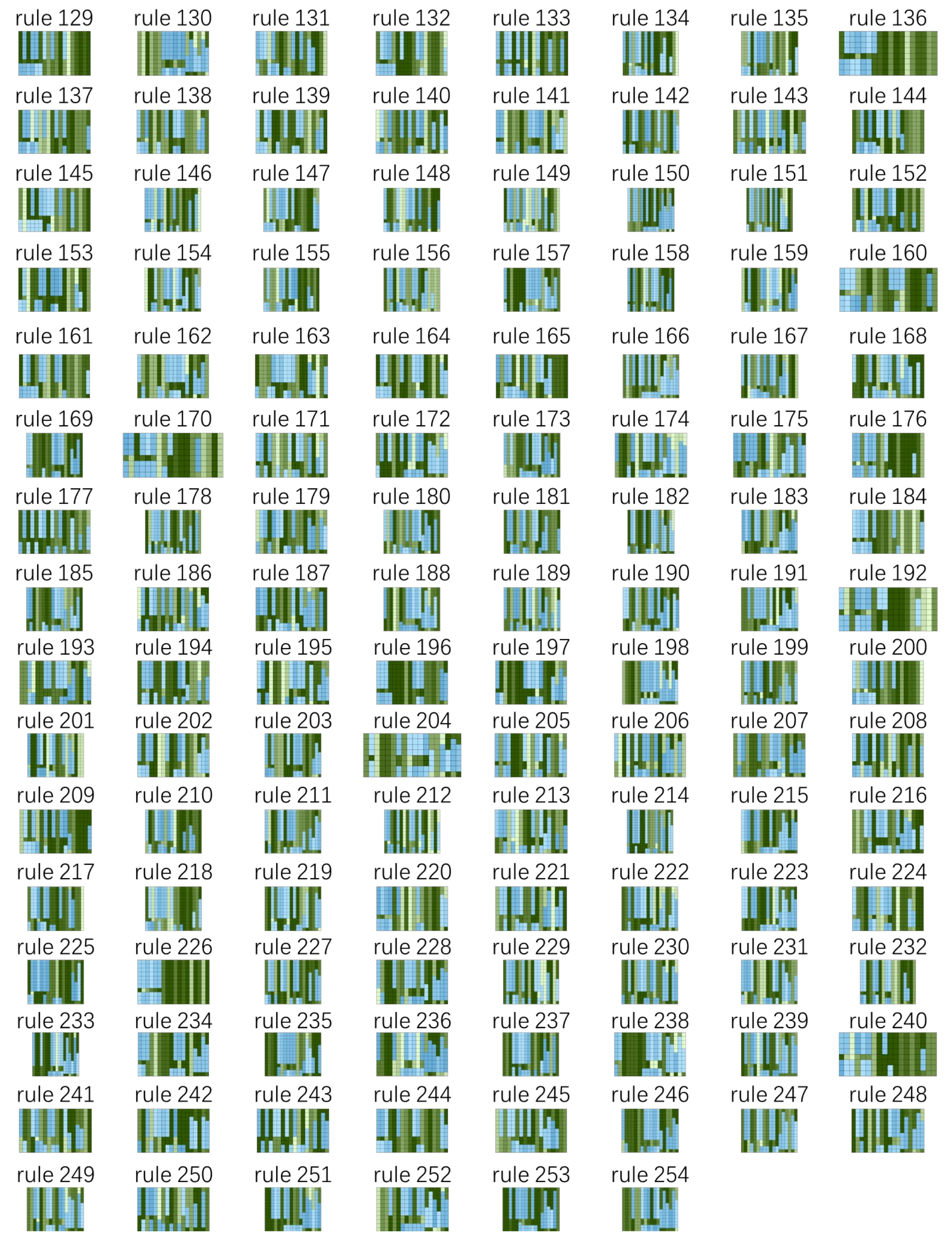

Supplementary Figure 16. Memristors evolution diagram for entire rule of 1D elementary cellular automata (Rule 129-254).

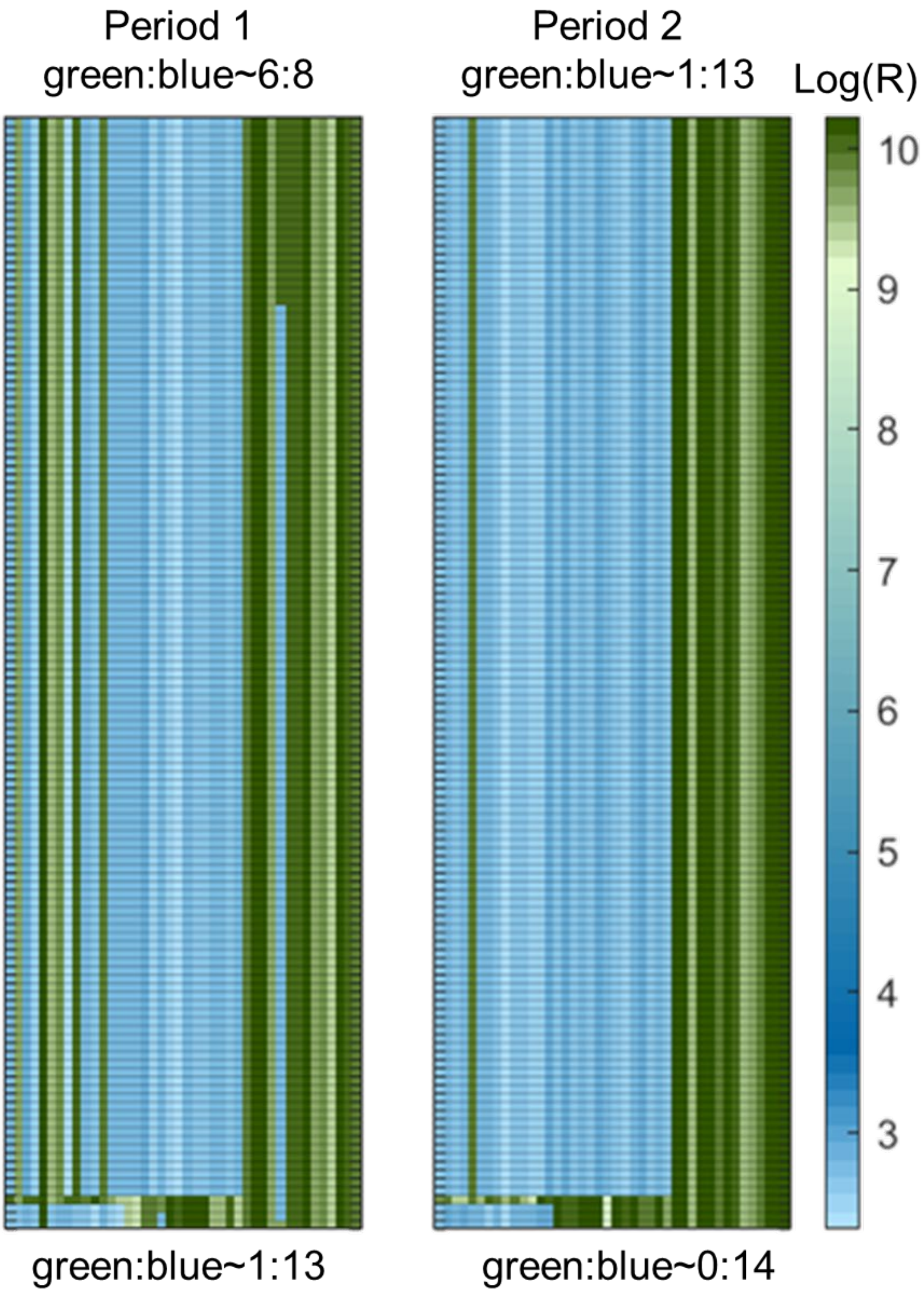

Supplementary Figure 17. Memristors evolution diagram for majority classification algorithm with 14 input. The initial input is 01001110100100. After two periods, all cells have been converted to ‘0’ state, which authenticate the algorithm.

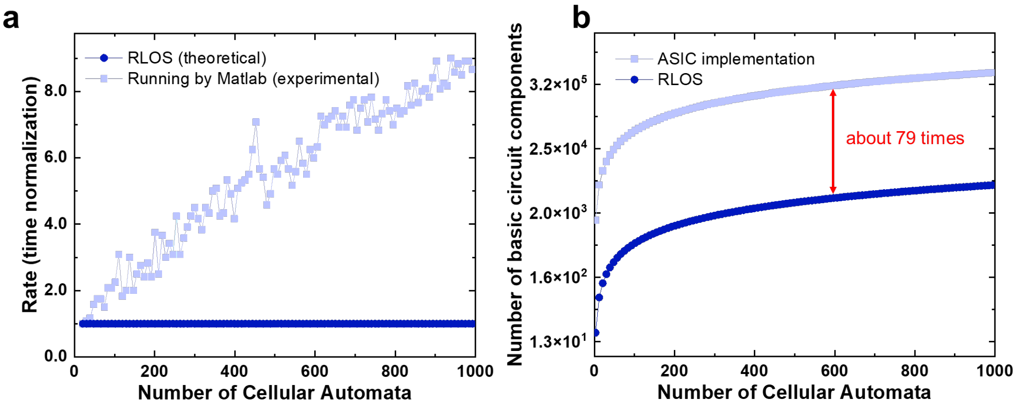

Supplementary Figure 18. The comparison between RLOS and conventional implementation. (a) The computing time comparison between our work and matlab implementation. (b) The hardware cost comparison between our work and ASIC implement.

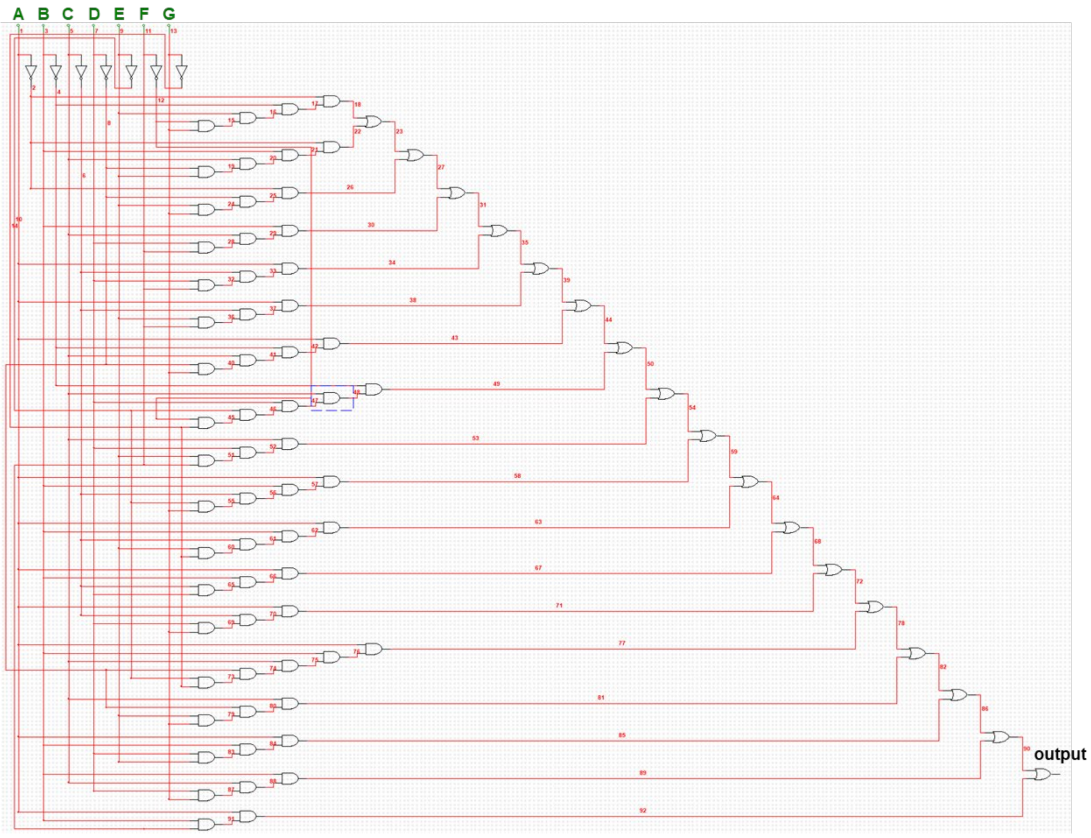

Supplementary Figure 19. Rule Switching Logic unit for Majority classification algorithm based on 1D CA. (Rule: 0504058705000f77037755837bffb77f[3], corresponding logic expression: A'B'EF'G + A'BCD'E + A'D'EG + BCDF + AC'DF

+ AC’EF + AB’CD’G + B’CDE’F’G’ + CDEF + ABC’E’G + ABC’EG’+ ABC’D + AC’DG + ABCD’E’G’ + CD’EG + ABDE + BCDG + ABF. Components number:86).

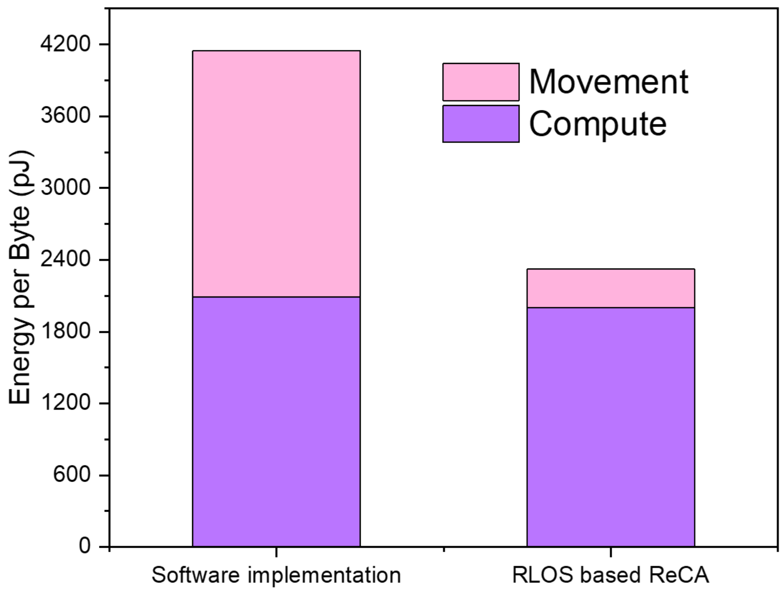

Supplementary Figure 20. Comparing estimation data movement of realization digital recognition between software and RLOS.

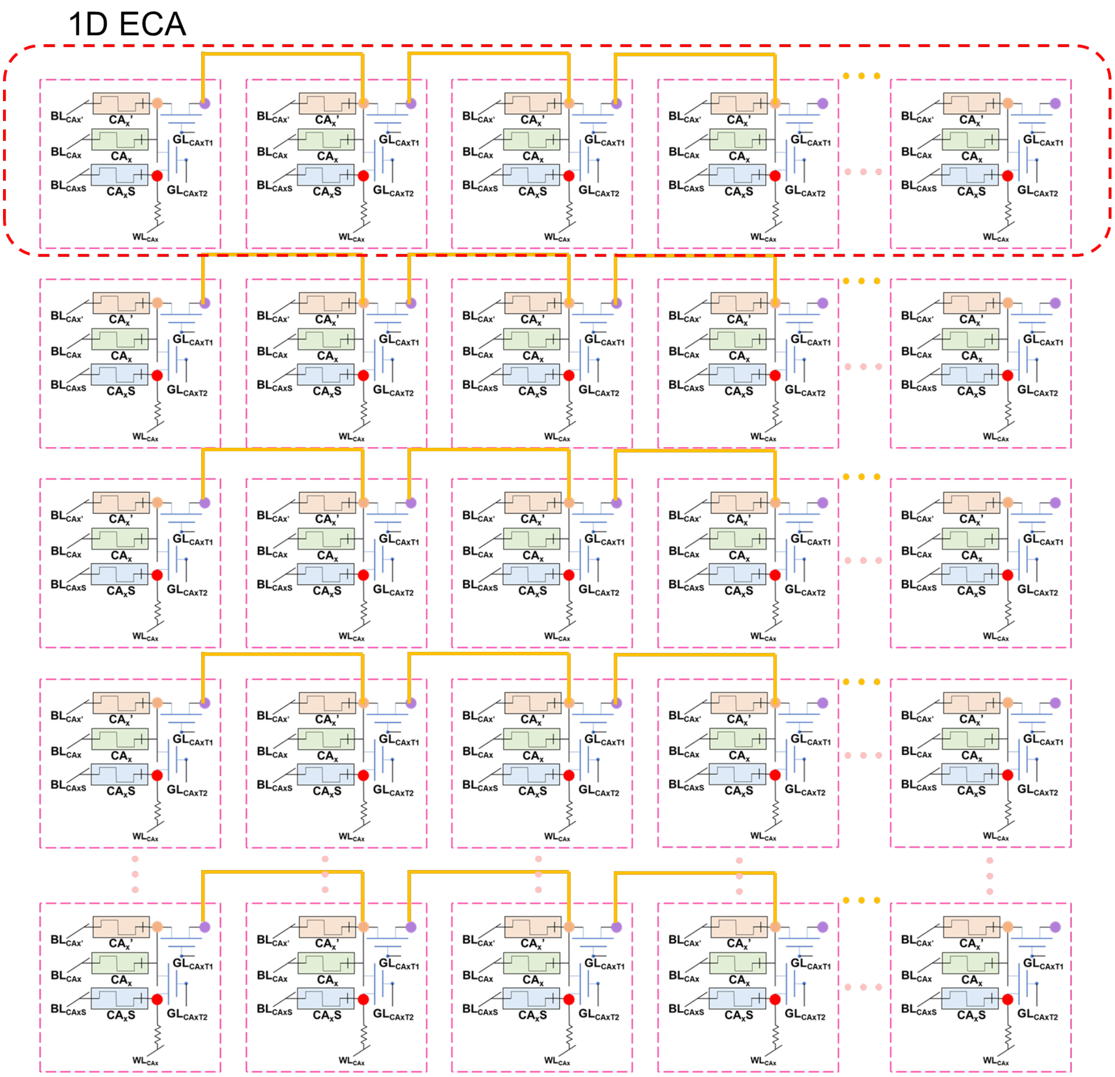

Supplementary Figure 21. The schematic of the first layer of circuits for CA operation in ReCA.

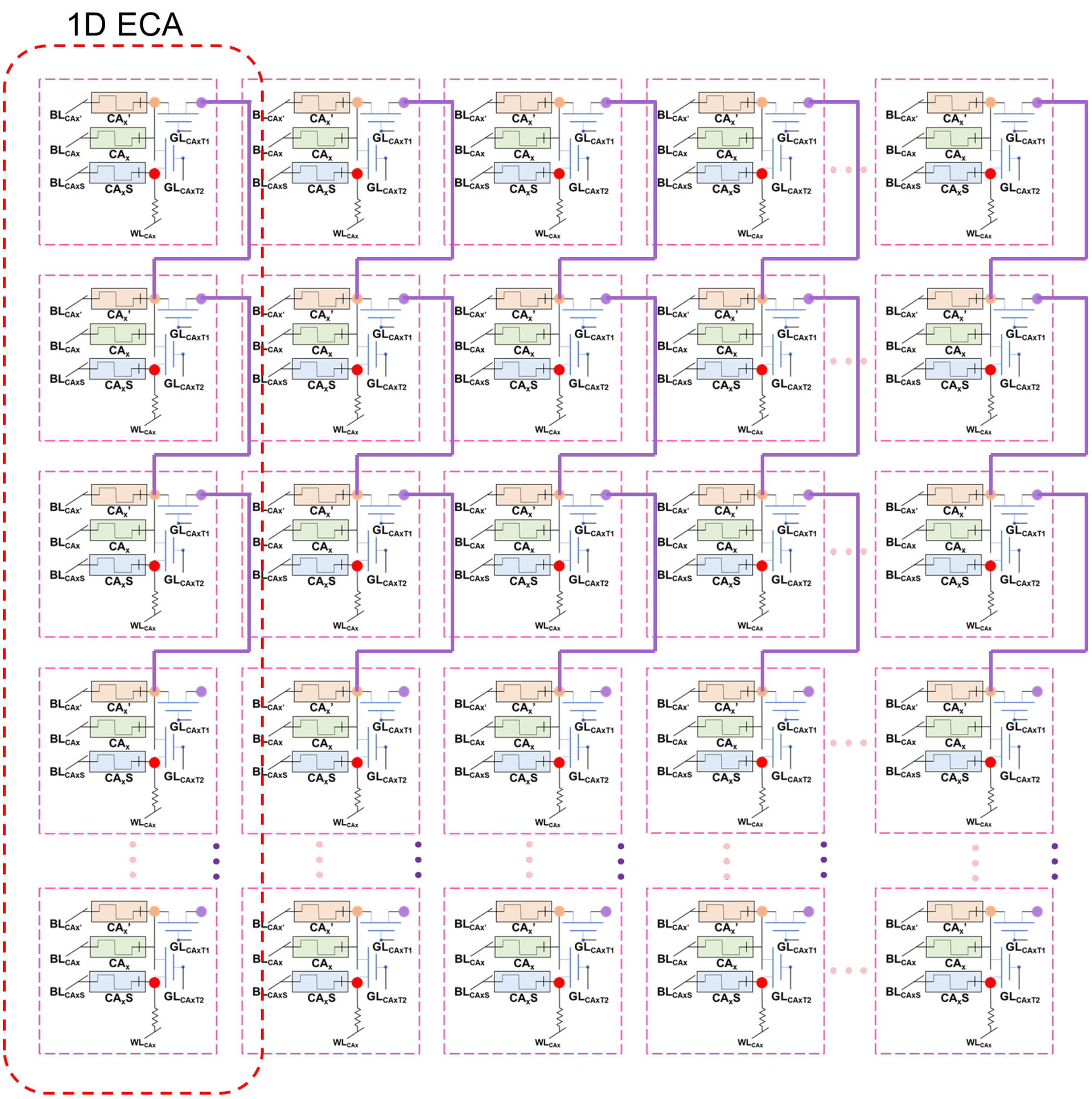

Supplementary Figure 22. The schematic of the second layer of circuits for CA operation in ReCA.

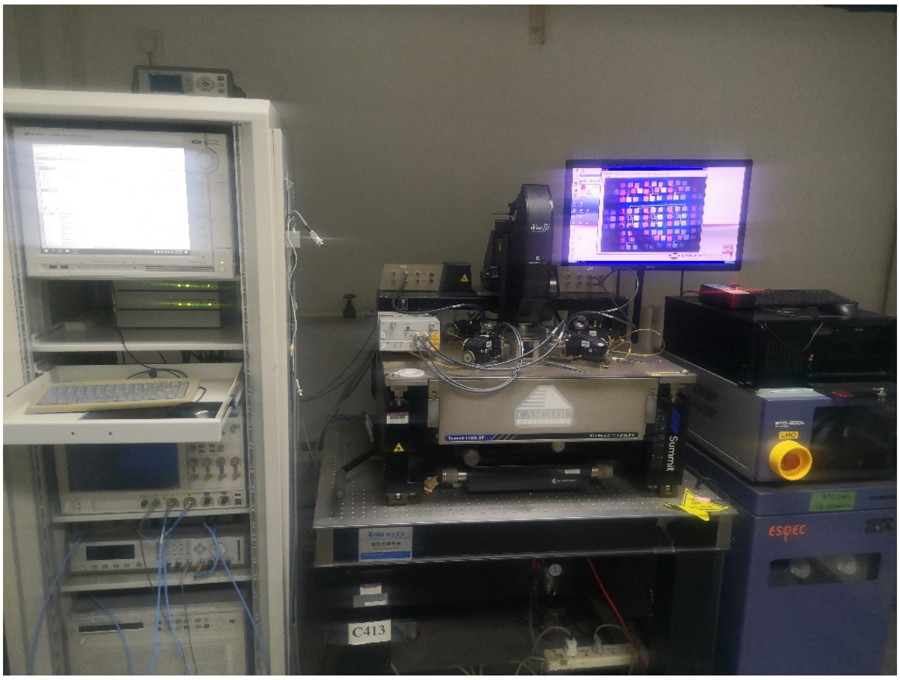

Supplementary Figure 23. The image of test equipment.

Supplementary Table 1. The comparisons of hardware cost and operation cost between cross-bar structure and RLOS, where n is the number of the cellular.

	Number of memristors	Number of transistors	Number of operations
Cross-bar	7	7	2n+11
RLOS	3	2	13

Supplementary Table 2. The logic operation of majority classification based on cellular automata.

	Logic Operation		Logic Operation		Logic Operation
1	S = NAND(A’,B’,E,F,G)	2	S = NAND(A’,B,C,D’,E)	3	S = NAND(A’,D’,E,G)
4	S= NAND(B,C,D,F)	5	S = NAND(A,C’,D,F)	6	S = NAND(A,C’,E,F)
7	S =NAND(A,B’,C,D’,G)	8	S = NAND(B’,C,D,E’,F’,G’)	9	S = NAND(C,D,E,F)
10	S = NAND(A,B,C’,E’,G)	11	S = NAND(A,B,C’,E,G’)	12	S = NAND(A,B,C’,D)
13	S = NAND(A,C’,D,G)	14	S = NAND(A,B,C,D’,E’,G’)	15	S = NAND(C,D’,E,G)
16	S = NAND(A,B,D,E)	17	S = NAND(B,C,D,G)	18	S = NAND(A,B,F)

Supplementary Table 3. Comparison with different technology to

implementation CA

	FPGA	Crossbar	RLOS
Number of memristors	0	7	3
Number of transistors	6~30	7	2
Number of operations	1	2n+11	13
Energy	~0.2174nW/cellular	~0.4561pW/cellular	~0.1954pW/cellular
Area efficiency	~180F2	~70F2	~63F2

Supplementary Table 4. Comparison of different processing in memory (PIM) technologies

Basic device	Basic structure	Structure	Foundational algorithm	reference
RRAM	2T3R	RLOS	Cellular automata	This work
RRAM	1T1R	Crossbar	Multiply-accumulate, basic logic	[4]
FeRAM	1T1C FeRAM, FeRAM	Crossbar	Multiply-accumulate, X(N)OR	[5, 6]
SRAM	S6T, 10T, T8T, 6T, 8T	column-based dot-product circuit, PIM macro array	Multiply-accumulate, addition, subtraction, bitwise	[4]
DRAM	3T1C, 1T1C-NOR/MIX, 1T1C-ADDER	PIM macro array	Multiply-accumulate, addition, subtraction, bitwise, shifting	[4]
Flash	2T2F	Crossbar	Multiply-accumulate	[7]

Supplementary Note 1 – The innovation of RLOS

The innovation of our work lies in the proposal of RLOS based on processing in memory (PIM), which expands the foundational algorithms of PIM. **Supplementary Table 3** shows the basic algorithm of different PIM implementation. Our work marks the first time that the CA algorithm has been introduced into PIM.

To further strengthen our achievements, we have elaborated on the three dimensions of advantages of our works:

1. We successfully integrated memristors with 2D transistors (Ag/HfO_2/Pb+MoS_2 FET). Unlike conventional CMOS technology, which requires the transistor to be positioned at the bottom layer for doping, the 2D material used here can be

transferred onto any substrate while maintaining its functionality. As a result, the RRAM and 2D transistors can be fabricated in the same layer and our fabrication method is significantly more efficient. While CMOS-based processes involve 16 steps, our innovative approach necessitates only 9 steps.

2. We proposed the RLOS, which can perfectly match the CA rules and is compatible with the requirements of different lengths of rules. RLOS saves the cost of information transfer and duplication. For fair comparison, we also do same task based on FPGA, crossbar and RLOS. The specific comparison has been shown in **Supplementary Table 4**. RLOS displays lower hardware cost and lower power consumption. It is worth noting that the extremely low hardware cost of RLOS offers tremendous potential in future fields such as edge computing.
3. In terms of applications, our work is the first PIM with CA algorithm and CA is a distinguished model that can be used for the study of the behavior of the system and complex phenomena. CA not only conducts as mathematical computation models, but is also an effective medium to simulate the natural phenomena and nature system. When studying complex systems, cellular automata is an efficient computing platform for its self-replication, self-organizing characteristic. CA also has the feature of graphical computing, which the previously mentioned PIM technology does not possess. CA has been extensively investigated since its inception. It is noted that some transition rules of CA are equivalent to Turing machines, which means it can simulate any computable problem. The famous computer scientist Stephen Wolfram has said that "even though they might not be the story of the particular way to model a particular thing, they're super interesting in terms of the pure basic science of what's going on"[8]. The CA based architecture to compose the computer has also been proposed by many references[9]. Therefore, CA has great potential in future computing.

Supplementary Note 2 – Estimation of the R_C

We use the Kirchhoff Laws to estimate the divided voltage of memristor Y. Coupled with the limited conditions according to the logic rules, the value of R_c can be determined. In our estimation, the 'On' state transistor is approximated to resistance as R_{t1} and R_{t2}.

The resistance of memristors and auxiliary resistance R are written as the R_a, R_b, R_y and R_s, respectively. Where R_a, R_b, R_y can change to LRS or HRS.

Define:

$$R_{abt} = R_{t2} + \frac{R_b(R_{t1} + R_a)}{R_b + R_{t1} + R_a}$$

$$R_{abty} = \frac{R_y R_{abt}}{R_y + R_{abt}}$$

$$R_{ys} = \frac{R_y R_s}{R_y + R_s}$$

Based on the circuit in Supplementary Figure 10:

$$V_b = \left(V_2 \frac{R_{abty}}{R_s + R_{abty}} - V_1 \frac{R_{abt}}{R_{abt} + R_{ys}}\right) \frac{R_b(R_{t1} + R_a)}{(R_b + R_{t1} + R_a)R_{abt}}$$

$$V_a = V_b \frac{R_a}{R_a + R_{t1}}$$

$$V_y = V_2 \frac{R_{abty}}{R_s + R_{abty}} + V_1 \frac{R_{ys}}{R_{abt} + R_{ys}}$$

$$V_s = -V_2 \frac{R_{abty}}{R_s + R_{abty}} + V_1 \frac{R_{ys}}{R_{abt} + R_{ys}}$$

Then we get all the voltages.

If Y memristor should switch, the calculation results should satisfy $V_y > 1.8\,V$. If Y memristor do not switch, the calculation results should satisfy $|V_y| < 1.2\,V$. Moreover, in any condition, the calculation results should satisfy $|V_a| < 1.2\,V$, $|V_b| < 1.2\,V$. The results in Supplementary Figure 11 also satisfy these limitations.

Supplementary Note 3 – Logic operation of edge detection algorithm

The transition rule of edge detection algorithm based on CA can be defined as the following table[10]:

The sum of the nine cell values	9	8	7	6	5	4	3	2	1	0
The next state of center cell	0	1	1	1	0	0	0	0	0	0

The transition rules can be converted to logic formula as:

Y= ABCDEFG’ + ABCDEF’G + ABCDEF’H + ABCDEF’I + ABCDGH’I + ABCDE’FG + ABCDE’FH + ABCDE’FI + ABCDF’GH + ABCDF’HI + ABCEGH’I + ABCEF’GH + ABCEF’HI + ABCFGH’I + ABCD’EFG + ABCD’EFH + ABCD’EFI + ABCE’FGH + ABCE’FHI + ABCF’GHI + ABDEGH’I + ABDEF’GH + ABDEF’HI

+ ABDFGH’I + ABDE’FGH + ABDE’FHI + ABDF’GHI + ABEFGH’I + ABEF’GHI + ABC’DEFG + ABC’DEFH + ABC’DEFI + ABD’EFGH + ABD’EFHI + ABE’FGHI + ACDEGH’I + ACDEF’GH + ACDEF’HI + ACDFGH’I + ACDE’FGH + ACDE’FHI + ACDF’GHI + ACEFGH’I + ACEF’GHI + ACD’EFGH + ACD’EFHI + ACE’FGHI + ADEFGH’I + ADEF’GHI + ADE’FGHI + AB’CDEFG + AB’CDEFH + AB’CDEFI + AC’DEFGH + AC’DEFHI + AD’EFGHI + BCDEFGI’ + BCDEFH’I + BCDEF’GH + BCDEF’GI + BCDEF’HI + BCDE’FGH + BCDE’FGI + BCDE’FHI + BCDF’GHI + BCEF’GHI + BCD’EFGH + BCD’EFGI + BCD’EFHI + BCE’FGHI + BDEF’GHI + BDE’FGHI + BC’DEFGH + BC’DEFGI + BC’DEFHI + BD’EFGHI + CDEF’GHI + CDE’FGHI + CD’EFGHI + A’BCDEFH + B’CDEFGH + B’CDEFGI + B’CDEFHI + C’DEFGHI.

Therefore, each logic operation in one step has 84 rounds. And each step has 756 operations.

Supplementary References

1. Liu Y, Yang K, Wang X, Tian H, Ren T. Lower Power, Better Uniformity, and Stability CBRAM Enabled by Graphene Nanohole Interface Engineering. *IEEE Transactions on Electron Devices* **67**, 984-988 (2020).

2. Huang P*, et al.* Reconfigurable Nonvolatile Logic Operations in Resistance Switching Crossbar Array for Large-Scale Circuits. *Advanced Materials* **28**, 9758-9764 (2016).

3. Mitchell M, Crutchfield JP, Das R. Evolving cellular automata with genetic algorithms: A review of recent work. In: *Proceedings of the First international conference on evolutionary computation and its applications (EvCA'96)*). Moscow (1996).

4. Kim J-Y, Kim B, Kim TT-H. *Processing-in-memory for AI: From Circuits to Systems*. Springer Nature (2022).

5. Wang D*, et al.* Ultrathin Nitride Ferroic Memory with Large ON/OFF Ratios for Analog In-memory Computing. *Advanced Materials* **n/a**, 2210628 (2023).

6. Qiao W*, et al.* Non-volatile In Memory Dual-Row X(N)OR Operation with Write Back Circuit Based on 1T1C FeRAM. In: *2020 IEEE 15th International Conference on Solid-State & Integrated Circuit Technology (ICSICT)*) (2020).

7. Hayashikoshi M*, et al.* Processing In-Memory Architecture with On-Chip Transfer Learning Function for Compensating Characteristic Variation. In: *2020 IEEE International Memory Workshop (IMW)*) (2020).

8. Wolfram S. Two Different Directions: John Conway and Stephen Wolfram. In: *The*

Mathematical Artist: A Tribute To John Horton Conway). Springer (2022).

9. Zhirnov V, Cavin R, Leeming G, Galatsis K. An Assessment of Integrated Digital Cellular Automata Architectures. *Computer* **41**, 38-44 (2008).

10. Itoh M, Chua LO. DIFFERENCE EQUATIONS FOR CELLULAR AUTOMATA. *International Journal of Bifurcation and Chaos* **19**, 805-830 (2009).
