# Supplementary material for: Cellular automata imbedded memristor-based recirculated logic in-memory computing
Source: Nat Commun. 2023 May 10;14:2695. doi: 10.1038/s41467-023-38299-7 (PMC10172358; doi:10.1038/s41467-023-38299-7)
Supplement: Supplementary file 2 — Description of Additional Supplementary Files [file 41467_2023_38299_MOESM2_ESM.docx]

File Name: Supplementary Data 1
Description: Supplementary Data 1. The logic expression of elementary cellular automata rules (1-254).
